# Design and crystal structure of a native-like HIV-1 envelope trimer that engages multiple broadly neutralizing antibody precursors in vivo

**DOI:** 10.1084/jem.20161160

**Published:** 2017-09-04

**Authors:** Max Medina-Ramírez, Fernando Garces, Amelia Escolano, Patrick Skog, Steven W. de Taeye, Ivan Del Moral-Sanchez, Andrew T. McGuire, Anila Yasmeen, Anna-Janina Behrens, Gabriel Ozorowski, Tom L.G.M. van den Kerkhof, Natalia T. Freund, Pia Dosenovic, Yuanzi Hua, Alexander D. Gitlin, Albert Cupo, Patricia van der Woude, Michael Golabek, Kwinten Sliepen, Tanya Blane, Neeltje Kootstra, Mariëlle J. van Breemen, Laura K. Pritchard, Robyn L. Stanfield, Max Crispin, Andrew B. Ward, Leonidas Stamatatos, Per Johan Klasse, John P. Moore, David Nemazee, Michel C. Nussenzweig, Ian A. Wilson, Rogier W. Sanders

**Affiliations:** 1 Department of Medical Microbiology, Academic Medical Center, University of Amsterdam, Amsterdam, Netherlands; 2 Department of Experimental Immunology, Academic Medical Center, University of Amsterdam, Amsterdam, Netherlands; 3 Department of Integrative Structural and Computational Biology, Scripps CHAVI-ID, IAVI Neutralizing Antibody Center and Collaboration for AIDS Vaccine Discovery (CAVD), The Scripps Research Institute, La Jolla, CA; 4 Department of Immunology and Microbiology, Scripps CHAVI-ID, The Scripps Research Institute, La Jolla, CA; 5 Laboratory of Molecular Immunology, The Rockefeller University, New York, NY; 6 Seattle Biomedical Research Institute, Seattle, WA; 7 Department of Microbiology and Immunology, Weill Medical College of Cornell University, New York, NY; 8 Oxford Glycobiology Institute, Department of Biochemistry, University of Oxford, Oxford, England, UK; 9 Howard Hughes Medical Institute, The Rockefeller University, New York, NY

## Abstract

Induction of broadly neutralizing antibodies (bNAbs) by HIV-1 envelope glycoprotein immunogens would be a major advance toward an effective vaccine. A critical step in this process is the activation of naive B cells expressing germline (gl) antibody precursors that have the potential to evolve into bNAbs. Here, we reengineered the BG505 SOSIP.664 glycoprotein to engage gl precursors of bNAbs that target either the trimer apex or the CD4-binding site. The resulting BG505 SOSIP.v4.1-GT1 trimer binds multiple bNAb gl precursors in vitro. Immunization experiments in knock-in mice expressing gl-VRC01 or gl-PGT121 show that this trimer activates B cells in vivo, resulting in the secretion of specific antibodies into the sera. A crystal structure of the gl-targeting trimer at 3.2-Å resolution in complex with neutralizing antibodies 35O22 and 9H+109L reveals a native-like conformation and the successful incorporation of design features associated with binding of multiple gl-bNAb precursors.

## Abstract (precis)

Induction of broadly neutralizing antibodies (bNAbs) to HIV would be a major advance toward an effective vaccine. A critical step in this process is the activation of naive B cells expressing bNAb precursors. Medina-Ramírez et al. developed a BG505 SOSIP.v4.1-GT1 trimer that activates bNAb precursors in vitro and in vivo.

## Introduction

An effective HIV vaccine will likely require the elicitation of protective titers of broadly neutralizing antibodies (NAbs [bNAbs]). The envelope glycoprotein (Env) on the virion surface is the only relevant target for bNAbs and, hence, is the main focus for antibody-based vaccine strategies. Approximately 30%–50% of infected individuals eventually develop bNAbs (Mascola and Haynes, 2013; van Gils and Sanders, 2013; Hraber et al., 2014; Burton and Mascola, 2015), and passive immunization studies have shown that various bNAbs can protect macaques from experimental challenge (Hessell et al., 2009; Barouch et al., 2013; Shingai et al., 2014; Gautam et al., 2016). However, it has not yet been possible to induce bNAbs by vaccination. Even eliciting NAbs with narrow specificity against neutralization-resistant (Tier-2) primary viruses has been challenging but is nevertheless possible (Sanders et al., 2015; Escolano et al., 2017; Sanders and Moore, 2017).

The Env spike on HIV-1 virions is a metastable complex consisting of three gp120 and three gp41 subunits associated through noncovalent interactions. Soluble trimers of the SOSIP design (de Taeye et al., 2016; Sanders and Moore, 2017) that faithfully mimic the native spike have yielded valuable insights into the structural details of how Env functions and the bNAb epitopes it presents (Ward and Wilson, 2017). SOSIP trimers have induced strong and consistent autologous Tier-2 NAb responses in rabbits and somewhat weaker responses in macaques (de Taeye et al., 2015; Sanders et al., 2015; Klasse et al., 2016). A major goal is now to devise a strategy to broaden these narrow specificity NAb responses into ones resembling bNAbs. To develop more sophisticated vaccination regimens will require combining our increasing knowledge of Env structure with an understanding of bNAb development.

During HIV-1 infection, bNAbs usually emerge over time from an initial, narrowly focused, autologous NAb response to transmitted/founder viruses that are susceptible to germline (gl)–bNAbs binding (Bonsignori et al., 2017). This process requires high levels of somatic mutation (Escolano et al., 2017) mediated by multiple cycles of viral escape from antibody pressure generating new variants that, in turn, drive additional antibody affinity maturation (Liao et al., 2013; Doria-Rose et al., 2014). Can Env immunogens be designed to mimic this process (Haynes et al., 2012; Klein et al., 2013a; Medina-Ramírez et al., 2017; Sanders and Moore, 2017)? To do so would require specific targeting and activation of B cell lineages that could eventually evolve into bNAb-producing clones. One approach involves engineering an immunogen to recognize the gl forms of bNAbs and thereby prime specific B cell lineages (Haynes et al., 2012; Ota et al., 2012; Jardine et al., 2013, 2015, 2016; Klein et al., 2013b; Dosenovic et al., 2015; Escolano et al., 2016; McGuire et al., 2016; Scharf et al., 2016; Steichen et al., 2016; Stamatatos et al., 2017). Boosting with additional immunogens to guide the affinity maturation pathway may then yield NAbs with the required breadth and potency (Bonsignori et al., 2017; Escolano et al., 2017; Stamatatos et al., 2017). The critical priming immunogen should, therefore, activate naive B cells expressing at least one potential bNAb precursor, preferably several. The gl precursors for several bNAbs have been inferred by sequence analysis, providing templates for guiding immunogen design (Pancera et al., 2010; Bonsignori et al., 2011; Scheid et al., 2011; Jardine et al., 2013; Doria-Rose et al., 2014; Sliepen et al., 2015).

A bNAb epitope cluster of interest is the CD4-binding site (CD4bs). The CD4 receptor and several subfamilies of bNAbs bind to overlapping epitopes on both gp120 monomers and native-like trimers. However, many antibodies that recognize CD4bs-associated epitopes on the outer domain of the gp120 monomer cannot do so on the trimer because of topological constraints imposed by the trimeric architecture. This subset of CD4bs antibodies is non-neutralizing (i.e., non-NAbs) for Tier-2 viruses (Chen et al., 2009).

The VRC01 class of bNAbs, which includes VRC01 and 3BNC60, epitomizes both the potential of the CD4bs and the challenges associated with the design of gl-targeting immunogens for eliciting such antibodies. The presentation of the epitopes for these potent bNAbs on both gp120 monomer and native trimer is now well understood at the structural level (Wu et al., 2011; Kong et al., 2016; Scharf et al., 2016). One key finding is how *N*-linked glycans in the loop D and V5 regions of gp120 impede binding of gl-bNAbs to the CD4bs (McGuire et al., 2013; Gristick et al., 2016; Kong et al., 2016). Thus, whereas the mature VRC01 and 3BNC60 bNAbs bind Env proteins with high affinity, the corresponding gl-bNAbs do not (Zhou et al., 2010; Scheid et al., 2011; Klein et al., 2013a). An unmodified Env immunogen would not, therefore, be likely to trigger the induction of these bNAb lineages. Structure-guided design has successfully produced Env-based proteins with increased affinity for gl-bNAbs of the VRC01 class, designated eOD-GT6/8 and 426c.TM4ΔV1-V3 (Jardine et al., 2013, 2016; McGuire et al., 2013, 2016). These immunogens were able to activate antibody responses in knock-in mice engineered to express the gl precursors of VRC01 or 3BNC60 but did not induce bNAbs (Dosenovic et al., 2015; Jardine et al., 2015; McGuire et al., 2016; Tian et al., 2016). Under the same conditions, native-like BG505 SOSIP.664 trimers did not initiate gl-VRC01 or gl-3BNC60 antibody lineages, which is consistent with their nonreactivity with these bNAb precursors in vitro (Dosenovic et al., 2015; Sliepen et al., 2015). However, when the same trimers were tested in knock-in mice transgenic for the mature 3BNC60 heavy chain, they selected an appropriate light chain from the antibody repertoire, which enabled induction of NAbs with some breadth (Dosenovic et al., 2015). These observations underpin our hypothesis that an engineered trimer that has an appropriate affinity for a gl-bNAb could initiate a B cell lineage that can be guided toward evolution of bNAbs by boosting with one or more rationally chosen Env trimers.

Another suitable target for gl-targeting Env immunogen design is the Env trimer apex that is recognized by bNAbs such as CH01, PG9/PG16, PGT145/PGDM1400, and VRC26 (Walker et al., 2009; Bonsignori et al., 2011; Doria-Rose et al., 2014; Sok et al., 2014). The trimer-apex epitopes are attractive vaccine design targets because apex-directed bNAbs derived from several different gl genes emerge comparatively early and frequently during HIV-1 infection. Moreover, although the latter bNAbs require high levels of somatic hypermutation for optimal breadth and potency, the extent of mutation is lower than for VRC01-class antibodies (Walker et al., 2009, 2011; West et al., 2012; Klein et al., 2013a; Doria-Rose et al., 2014). These properties suggest that inducing similar bNAbs in humans by vaccination may be easier than inducing bNAbs against other epitopes. As trimer-apex bNAbs recognize gp120-V2 epitopes that are either highly influenced by, or completely dependent on, the quaternary structure of the trimer, a trimer-based immunogen is most likely required to initiate these lineages. Some native-like trimers, including BG505 SOSIP.664, can engage trimer-apex gl-bNAbs (Andrabi et al., 2015; Sliepen et al., 2015; Gorman et al., 2016), providing a strong foundation for structure-guided design improvements to yield higher affinity immunogens.

Here, we describe an engineered trimer variant, BG505 SOSIP.v4.1-GT1 (gl-targeting trimer version 1), with improved capacity for binding gl-bNAbs that target the trimer-apex and the CD4bs epitopes.

## Results

### Design of the BG505 SOSIP.v4.1-GT1 trimer

Our goal was to engineer a variant of the BG505 SOSIP.664 Env trimer with enhanced binding to inferred gl-bNAbs, including those targeting the CD4bs and the V1V2-apex (Table S1). To remove impediments to trimer binding of CD4bs gl-bNAbs, we eliminated potential *N*-linked glycosylation sites (PNGSs) in loop D and V5 via N276D and N462D substitutions, respectively (Li et al., 2011; Wu et al., 2011; Jardine et al., 2013; Joyce et al., 2013; McGuire et al., 2013). We also removed two additional PNGSs at positions 386 in the mannose patch (via N386D; Sanders et al., 2008; Li et al., 2011; Jardine et al., 2013) and at 197 in the bridging sheet (via S199A; Li et al., 2011). Finally, we created a favorable contact between loop D and the VRC01 light chain (via T278R) and also stabilized loop 5 (via G471S; Jardine et al., 2013).

To obtain insights into V1V2-apex epitopes, we measured the ability of gl versions of PG9, PG16, and PGT145 (and, for comparison, the mature bNAbs) to neutralize a panel of 30 viruses and then analyzed the V2 sequences of the neutralization-sensitive and -resistant subgroups (Table S2). The panel included a BG505.T332N-LAI chimeric infectious molecular clone derived from the BG505 isolate (unpublished data), as well as BL035 and Q23 clade A Env-pseudotyped viruses that have epidemiological and genetic links to BG505 (Table S2; Neilson et al., 1999; Wu et al., 2006; Bonsignori et al., 2011). The remaining viruses were 27 clade B clinical isolates obtained 1–12 mo after infection from patients enrolled in the Amsterdam Cohort Studies on HIV/AIDS (ACS) that developed moderate to strong neutralization breadth (Table S2; Euler et al., 2010, 2011; van den Kerkhof et al., 2013; Lynch et al., 2015). The rationale for choosing these 27 viruses was that early Env sequences from patients whose neutralization response broadened over time might be markedly more reactive with gl-bNAbs than Env sequences shaped by the antibody response in chronically infected people (Liao et al., 2013; Doria-Rose et al., 2014).

BG505.T332N-LAI infectious molecular clone was resistant to all three gl-bNAbs at the maximum concentration tested, while BL035 was neutralized (>50% inhibition) by gl-PG9 and gl-PG16, and Q23 was sensitive to all three. Four of the ACS clade B viruses (D16928, D12950, H19829, and H19793) were neutralized by all three gl-bNAbs and six more by two of them (Table S2). We then aligned the Env V2 amino acid sequences from the viruses neutralized by two or three gl-bNAbs to identify possible determinants of gl-bNAb engagement. Relevant sequence changes were then introduced, alone or in combination, into the BG505 sequence to construct new SOSIP trimer variants for assessment of gl-bNAb reactivity (see below). Among these changes was a seven-residue deletion in the gl-bNAb-sensitive Q23 virus compared with the resistant BG505 virus, also from clade A (Tables S3 and S4). We also selected two BL035 residues (Y173H and S174A) and three Q23 residues (K169R, V181I, and Q183P; Tables S3 and S4). Several other potential influences on gl-bNAb reactivity were identified when the ACS clade B V2 sequences were analyzed, but only one had a beneficial effect when tested experimentally, specifically the introduction of an NTS sequon (G188N, N189T, and E190S) from the D12950 sequence (Table S3 and not depicted). We also noted that a non-BG505 peptide that had nanomolar affinity for gl-PG9 and gl-CH01 had a R178K change relative to the BG505 sequence (Aussedat et al., 2013).

**Figure 1.**
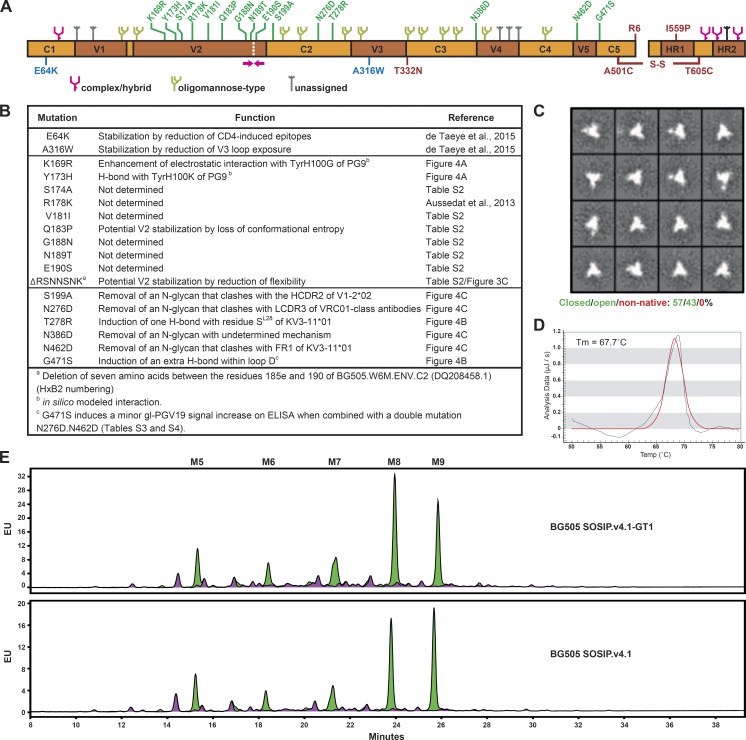
**Design and biophysical properties of a germline-targeting SOSIP trimer.** (A) Schematic of the BG505 SOSIP.v.4.1-GT1 construct (also referred to as GT1 trimer). The constant (C1–C5) and variable (V1–V5) regions in gp120 and the HR1 and HR2 regions in gp41 are indicated. The SOSIP mutations as well as the added N332 PNGS are shown in red. The E64K and A316W stabilizing mutations introduced to the SOSIP.664 construct to create SOSIP.v4.1 are indicated in blue. The mutations then introduced to SOSIP.v4.1 to induce gl-bNAb binding are indicated in green. The approximate position of a seven amino acid deletion is indicated with magenta arrows and a white dashed line. The glycan composition is adapted from Behrens et al. (2016). (B) Overview of the 18 changes introduced to BG505 SOSIP.664 to obtain SOSIP.v4.1-GT1. (C) NS-EM analyses of the GT1 trimer purified by PGT145. The 2D class averages are shown. On the basis of loop movement, compactness, and angles between individual protomers, the trimers are classified as closed native-like, partially open native-like, or nonnative (Pugach et al., 2015). The proportion of each class is indicated. (D) DSC analysis of the GT1 trimer purified with PGT145. The *T*_m_ value is indicated. (E) Glycan profiles of PGT145-purified trimer variants as determined by hydrophilic interaction liquid chromatography–ultraperformance liquid chromatography. The percentages of Man_5-9_GlcNAc_2_ glycans (M5-M9; shown in green), as a proportion of the total glycan population, are listed in Table S6.

Collectively, sequence changes relevant to the trimer-apex and CD4bs epitopes are outlined in Fig. 1 (A and B). Various changes were introduced, singly or in combination, into D7324- or His-tagged BG505 SOSIP constructs. The designs also incorporated one or both of the A316W and E64K substitutions, which confer additional stability to trimers designated as SOSIP.v4.1 when both substitutions are combined (Fig. 1, A and B; and Tables S3, S4, and S5; de Taeye et al., 2015). The variant trimers were expressed by transient transfection of human embryonic kidney 293T (HEK293T) cells, and the unpurified culture supernatants were used in a D7324 or a Ni-NTA/His-tag capture ELISA, as appropriate, to obtain preliminary estimates of expression levels (2G12 binding), native-like trimer formation (PGT145 binding), and gl-bNAb reactivity (Tables S3 and S4). The most promising new constructs were then further purified by PGT145 for more extensive evaluation (Table S5). The final outcome was that the BG505 SOSIP.664 construct was modified by nine substitutions in V2 (K169R, Y173H, S174A, R178K, V181I, Q183P, G188N, N189T, and E190S), a seven-residue deletion in V2, six sequence changes around the CD4bs (S199A, N276D, T278R, N386D, N462D, and T471S), and the E64K and A316W substitutions for stability. The resulting trimers were expressed efficiently and bound several gl-bNAbs targeting both the trimer-apex and the CD4bs (Tables S3 and S4; see below). The construct including all the above 18 modifications is designated BG505 SOSIP.v4.1-GT1 or, for convenience, the GT1 trimer.

The PGT145-purified GT1 trimer was fully cleaved as assessed by BN-PAGE and reducing and nonreducing SDS-PAGE (not depicted) and predominantly native-like when visualized by negative-stain electron microscopy (NS-EM; Fig. 1 C). Its midpoint of thermal denaturation (*T*_m_), as assessed by differential scanning calorimetry (DSC), was 67.7°C (Fig. 1 D), which is almost identical to that of the BG505 SOSIP.664 prototype (Sanders et al., 2013). Finally, the glycan profile of the GT1 trimer was dominated by oligomannose glycans, similar to those of the parental SOSIP.664 and SOSIP.v4.1 trimers as well as native trimers on virions, but with a slightly higher ratio of Man_8_:Man_9_ (Fig. 1 E and Table S6; Behrens et al., 2016).

### BG505 SOSIP.v4.1-GT1 trimers bind multiple gl-bNAbs

**Figure 2.**
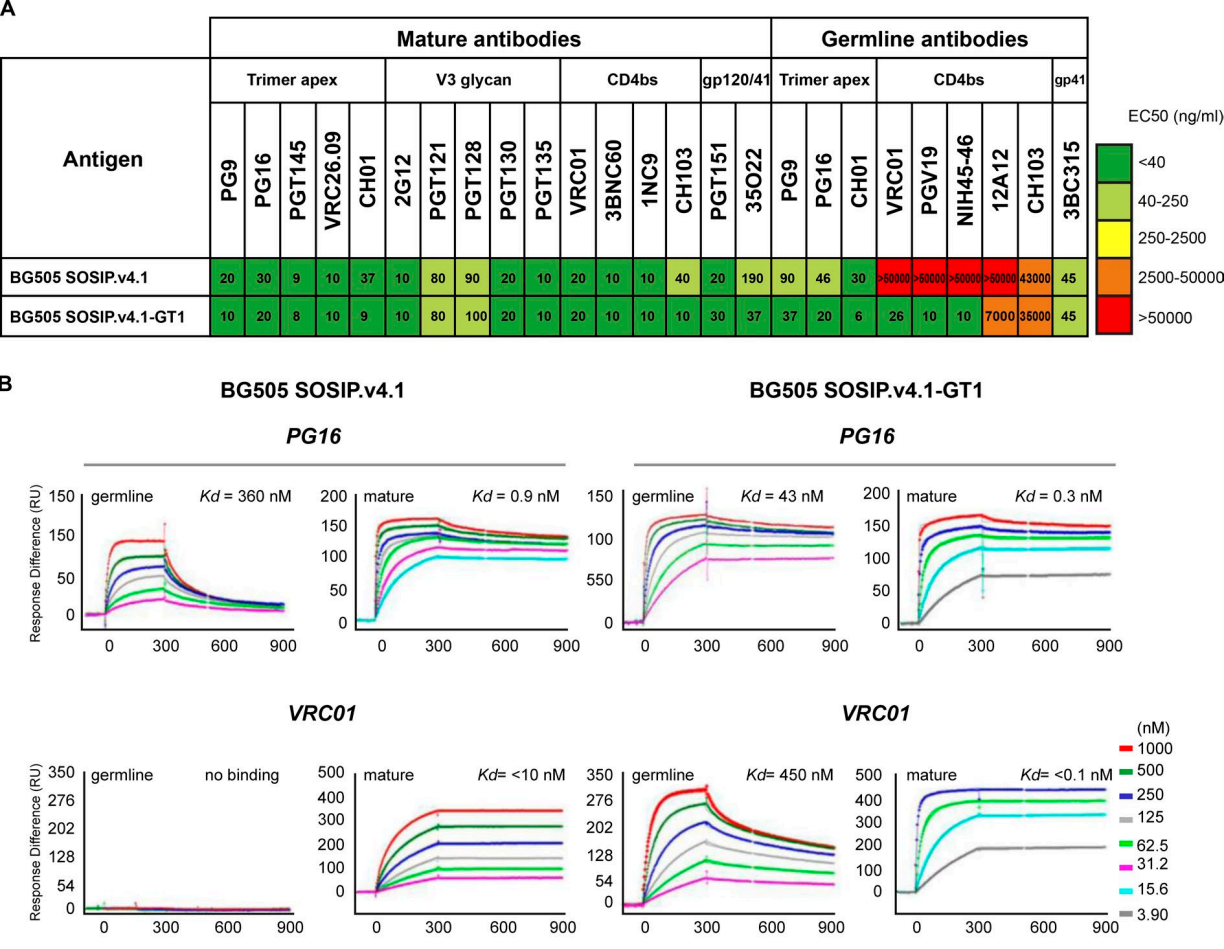
**Antigenicity of the BG505 SOSIP.v4.1-GT1 trimer with a panel of bNAbs and gl-bNAbs.** (A) Binding of bNAbs and gl-bNAbs to different SOSIP trimers was assessed by capture ELISA. Half maximal binding concentrations (EC_50_ [μg/ml]) are shown, and ranges in nanograms per milliliter are color coded. (B) Representative binding SPR curves of the binding of PG16 and VRC01 mature and germline versions to SOSIP.v4.1 and SOSIP.v4.1-GT1. The sensorgrams show the response (RU) over time (seconds). The association phase is 300 s, and the dissociation is followed over 600 s. Curves for concentration ranges (see inset) are shown in color with the modeled fits in black overlaid with the corresponding dissociation constant (*K_d_* = *K_d1_* for the monovalent initial interaction; see Table S7). SPR experiments were performed at least three times independently.

In a capture ELISA, PGT145-purified GT1 trimers bound several trimer-apex gl-bNAbs (gl-PG9, gl-PG16, and gl-CH01) more strongly (two- to fivefold) than did the SOSIP.v4.1 precursor (Fig. 2 A and Fig. S1 B). Three CD4bs-directed VRC01-class gl-bNAbs (gl-VRC01, gl-NIH45-46, and gl-PGV19) bound well to the GT1 trimers, and two others (gl-12A12 and gl-CH31) did so at an intermediate level, which contrasts with their undetectable binding to the unmodified trimers (Fig. 2 A and Fig. S1 B). We observed very weak binding of the CD4bs-directed gl-CH103 to the SOSIP.v4.1 and GT1 trimers on ELISA (Fig. S1 B), but only weak binding to GT1 by surface plasmon resonance (SPR; Fig. S2). gl-1NC9 or gl-3BNC60 binding was unmeasurable. There was no detectable, or only minimal binding, of the mature CD4bs-directed non-NAbs b6 and F105 to either trimer (Figs. S1 A and S3). We previously reported that gl-3BC315 (against a conformational epitope on gp41) bound to the unmodified BG505 SOSIP.664 trimer (Sliepen et al., 2015). The epitope for this gl-bNAb was preserved on the GT1 variant (Fig. 2 A and Fig. S1 B).

We analyzed antibody binding by SPR by using immobilized His-tagged trimers and antibodies (IgG) as the analyte and by applying a bivalent model to the data (Yasmeen et al., 2014). Both the mature and gl versions of PG16 had higher affinities (i.e., lower *K_d1_* values) for GT1 trimers than for SOSIP.v4.1; the extent of binding (stoichiometries; *S_m_* values) to GT1 was also greater than to SOSIP.v4.1 trimers for both versions of PG16 (Fig. 2 B and Table S7). The higher affinities (i.e., lower *K_d1_* values) were attributable to both higher on-rate and lower off-rate constants (*k_on1_* and *k_off1_*). Similarly, the mature versions of the CD4bs-specific bNAbs VRC01, 3BNC60, and CH103 had greater on-rate constants and extents of binding to GT1 than to SOSIP.v4.1 trimers, although their off-rate constants were too low to be determined (*k_off1_* < 10^−5^ [1/s]). The gl versions of these bNAbs did not bind detectably to SOSIP.v4.1 trimers but did bind to GT1, although only gl-VRC01 had an affinity strong enough to be quantified (Fig. S2 and Table S7). Another mature bNAb to the CD4bs, 1NC9, likewise had a higher on-rate, *k_on1_* (although also a higher *k_off1_* and lower affinity) and stoichiometry of binding to GT1 than to SOSIP.v4.1 trimers. However, the gl-1NC9 version failed to bind to any trimer. The CD4bs non-NAbs b6 and F105 reacted weakly with BG505 SOSIP.664 trimers but did not bind the SOSIP.v4.1 variant detectably (Fig. S3). We confirmed that b6 and F105 were also nonreactive with the GT1 trimer, which implies that the modifications did not adversely affect the geometry of its CD4bs and associated epitopes. Finally, the mature bNAb PGT121 bound strongly and with comparable affinities to its N332/V3-base epitope on the SOSIP.v4.1 and GT1 trimers, but its gl version bound to neither trimer (Table S7).

Thus, the SPR analyses showed that the modifications that created the GT1 trimer enabled or enhanced the binding of gl versions of PG16 and three CD4bs bNAbs (VRC01, CH103, and 3BNC60), in particular by improving their on-rate constants and stoichiometries, while also improving the binding of some mature bNAbs.

### Crystal structure of the BG505 SOSIP.v4.1-GT1 trimer

**Figure 3.**
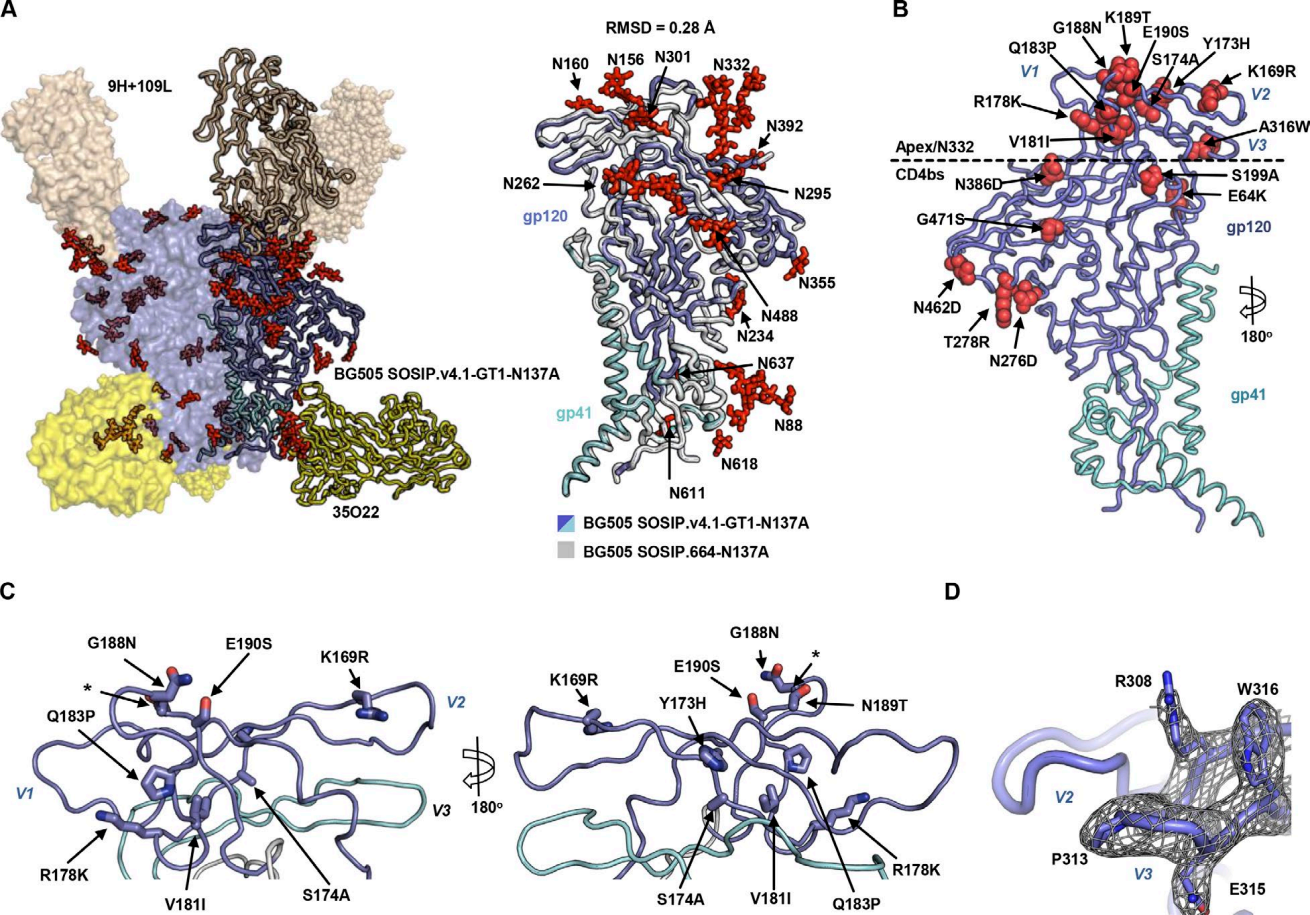
**Overall architecture of BG505 SOSIP.v4.1-GT1-N137A at 3.2 Å.** (A) Left: Side view of the trimer in complex with 9H+109L and 35O22 Fabs. The three protomers of the trimer complex are depicted as a surface representation (front left), spheres (back), and ribbon (front right), the latter with gp41 (in cyan) and gp120 (in blue). Each Env protomer (blue) is associated with one Fab 9H+109L (brown) and one Fab 35O22 (yellow). Glycans are shown in red sticks. Right: Structural alignment of one protomer (ribbon) from BG505 SOSIP.v4.1-GT1-N137A at 3.2 Å (gp120 in blue and gp41 in cyan) superimposed on one protomer of BG505 SOSIP.664-N137A (gray; PDB accession no. 5CEZ). The root-mean-square deviation (RMSD) is indicated, and *N*-linked glycans are shown and numbered by their respective Asn residues. (B) Ribbon representation of one protomer illustrating the mutations introduced to SOSIP.v4.1-GT1 to improve stability and enhance gl-bNAb-binding. (C) Zoomed-in 180° views of the apex region. Mutations are indicated with arrows, and the side chains are represented as sticks. The asterisk indicates the location of the truncated V2 loop after the seven amino acid deletion. (D) Detailed view of the V3 showing the A316W substitution with a 2Fo-Fc electron density map contoured at 1.0σ.

Inserting, deleting, or substituting individual residues could have ramifications on the overall protein conformation, including the reorganization or rearrangement of quaternary epitopes such as those found at the Env trimer apex. Here, we sought to investigate, at the atomic level, whether the 17 amino acid substitutions and seven-residue deletion in the GT1 trimer perturbed its structure compared with its SOSIP.664 prototype (Julien et al., 2013; Pancera et al., 2014; Garces et al., 2015). Guided by the 3.0-Å crystal structure of the BG505 SOSIP.664-N137A trimer (Garces et al., 2015), we removed the PNGS at position 137 (via N137A) from the GT1 construct for crystallization. The resulting trimer was combined with 35O22 Fab to stabilize the gp120-gp41 interface and promote crystal packing (Pancera et al., 2014), and with 9H+109L Fab, an N332/V3-base-directed antibody that binds with high affinity when the N137 glycan is absent (Garces et al., 2015; note that 9H is a putative heavy-chain precursor of the PGT121 family). We were then able to determine the crystal structure of GT1 at 3.2 Å resolution (Fig. 3 A).

A structural alignment of the GT1 and prototype SOSIP.664 trimers showed a Cα root-mean-square deviation of 0.28 Å (Table S8), indicating that the gl-targeting design changes did not substantially alter the native trimer conformation (Fig. 3 A). The structure allowed us to visualize the location of the engineered substitutions (Fig. 3, B and C) and thus supported the rationale for the design (see below). Of note is the extensive electron density for W316, which was introduced to decrease V3 mobility and increase trimer stability (Fig. 3 D; de Taeye et al., 2015). The side chain of W316 could possibly adopt more than one rotamer (which is difficult to define precisely at this resolution) but is clearly positioned between the side chains of R308 and Y318, providing a possible explanation for how it stabilizes the V3. Moreover, use of 9H+109L allowed comparison of its epitope and mode of binding with 3H+109L, a proposed precursor of 9H in the PGT121 heavy-chain lineage (Fig. S4 A; Garces et al., 2015). Both antibodies adopt the same angle of approach, and the glycans in and around their epitopes at positions N332, N301, and N156 have highly conserved conformations in the two structures (Fig. S4, A and B); the same is true of the conserved GDIR motif at the base of V3, a key component of the PGT121 and PGT128 epitopes (Fig. S4 B; Garces et al., 2014). A slight conformational change in the V1 tip (Fig. S4 B) might be attributed to the deletion of the N137 glycan, as previously observed (Garces et al., 2015).

### Models of the BG505 SOSIP.v4.1-GT1 trimer with VRC01-class gl-bNAbs and PG9

To understand in atomic detail how the engineered changes increase the affinity of the GT1 trimer for gl-bNAbs, we superimposed the structures of several Env proteins in complex with VRC01-class precursors onto the GT1 trimer structure. We also created an in silico model of the GT1 trimer + PG9 complex using information from the crystal structure of mature PG9 in complex with a scaffolded V1V2 domain from the ZM109 isolate (see below; McLellan et al., 2011).

**Figure 4.**
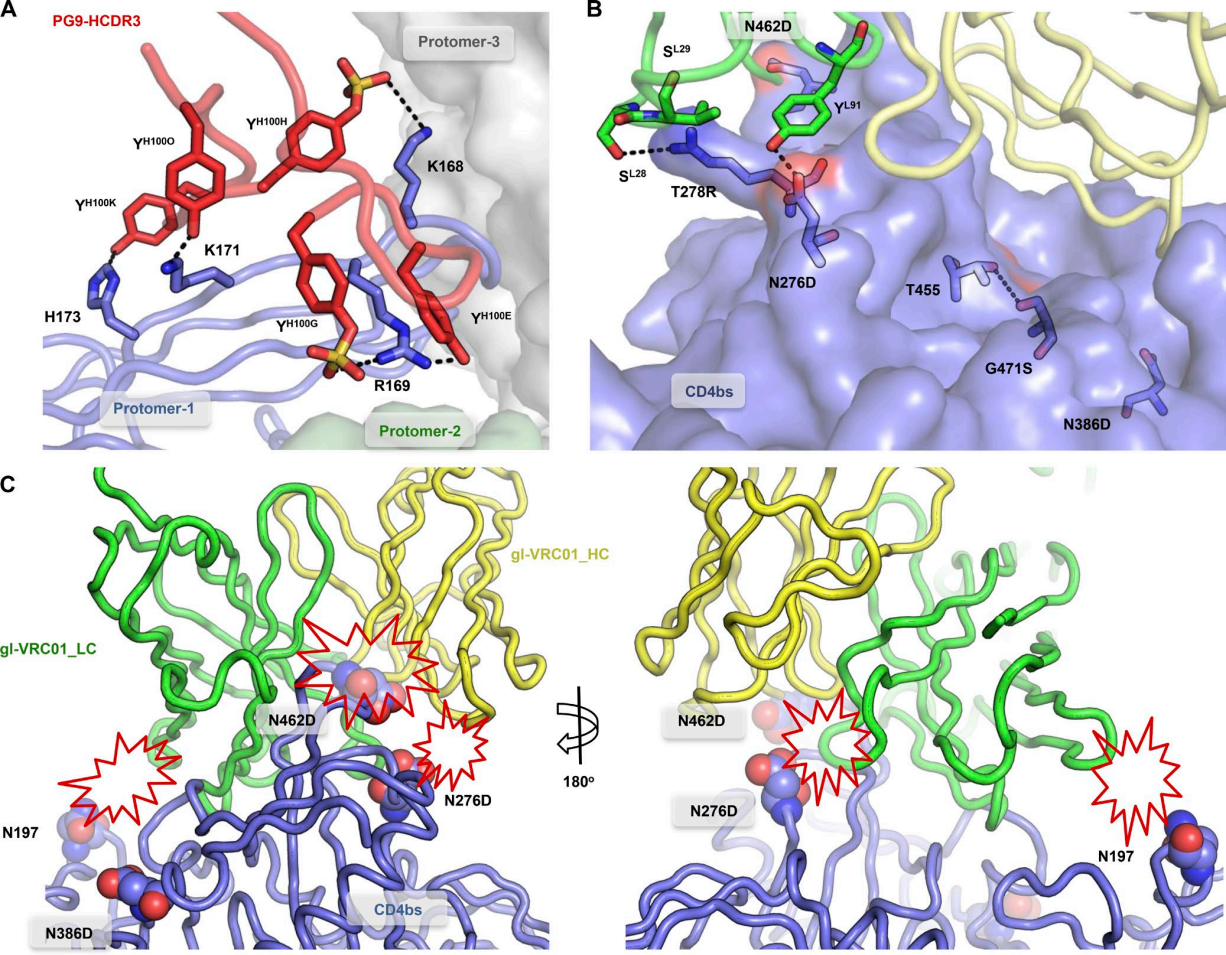
**Structural mechanism of germline engagement.** (A) Model of the interaction between PG9 HCDR3 (red) and V1V2 epitope of BG505 SOSIP.v4.1-GT1-N137A (blue). Relevant amino acid positions are indicated, and side chains are shown as blue sticks for Env and red sticks for HCDR3. Predicted interactions (<4 Å) between side chains are indicated with black dashed lines. (B) Model of interaction between the light chain of gl-VRC01 (green) and the loop D of SOSIP.v4.1-GT1-N137A (blue). Predicted interactions (<4 Å) between side chains are indicated with black dashed lines. The D462 residue, a substitution made to delete a possible obstructing glycan, is shown in the background (surface red). An intraprotomer H-bond (<4 Å) between T455 and S471 is indicated with black dashed lines. (C) Interaction between gl-VRC01 and the CD4bs, modeled in two different views. The positions of three PNGS in BG505 are indicated with spheres and their likely clashes with gl-VRCO1 light chain by red explosion shapes.

The superimposition of the eOD-GT6 + gl-VRC01 complex (Protein Data Bank [PDB] accession no. 4JPK; Jardine et al., 2013) onto the GT1 trimer structure confirms how removal of *N*-linked glycans at N197, N276, and N462 most likely reduce potential clashes with gl-VRC01 (Fig. 4 C). This outcome is consistent with the ELISA data (Table S3). The N276D substitution also allows formation of a hydrogen bond (H-bond) with the antibody TyrL91 (Fig. 4 B), the T278R change creates an additional contact with SerL28 (Fig. 4 B; Jardine et al., 2013), and G471S appears to have a stabilizing effect on the V5 loop by a facilitating a new intra-gp120 H-bond with Thr455 (Fig. 4 B). Superimposing the 426c.TMΔ1-3 gp120 + gl-NIH45-46 complex (PDB accession no. 5IGX; Scharf et al., 2013, 2016) onto the GT1 trimer highlights the extensive overlap between the contact residues of gl-VRC01 and gl-NIH45-46 (Fig. S4, C–E).

In the 1.8-Å crystal structure of the scaffolded ZM109 V1V2 domain with mature PG9 (PDB accession no. 3U2S), Lys169 (in V2 strand C) forms an H-bond with a sulfated tyrosine (Tys) at position 100G in HCDR3 (TysH100G; McLellan et al., 2011). Our in silico modeling suggests that Arg169 of the GT1 trimer can also form such an H-bond, but the guanidinium of Arg169 can form stronger electrostatic interactions with the sulfated TysH100G of PG9 (Woods et al., 2007). The V2 contact with TysH100G is important because the presence of the HCDR3 YYD-motif encoded by the gl D3-3*01 gene could help the GT1 trimer select antibodies that contain this motif (Andrabi et al., 2015). Furthermore, Arg169 might also form an additional H-bond with a neighboring (nonsulfated) TyrH100E residue (Fig. 4 A). These additional interactions might explain why the GT1 trimer has enhanced affinity for PG9 and gl-PG9. The model also sheds light on the Y173H substitution. In the PG9/ZM109-V1V2 reference complex, Asn173 forms an H-bond with TyrH100K (McLellan et al., 2011). In BG505 SOSIP.664, Tyr173 would clash with TyrH100K (not depicted), but the Y173H substitution in the GT1 trimer would eliminate this clash and enable an H-bond to form with TyrH100K (Fig. 4 A) in mature PG9 and also with TrpH100K in gl-PG9.

In crystal structures of BG505 SOSIP.664, which contain the full-length V2 loop, nine V2 residues starting from Asn185 and including two PNGS (PDB accession no. 4TVP; Julien et al., 2013; Pancera et al., 2014) are unresolved. We hypothesize that this flexible region inhibits interactions with HCDR3 of mature and precursor bNAbs, such as PG9, and therefore the seven amino acid deletion in this region of V2 in the GT1 trimer might alleviate that inhibition.

### In vitro and in vivo activation of B cells expressing gl-VRC01

**Figure 5.**
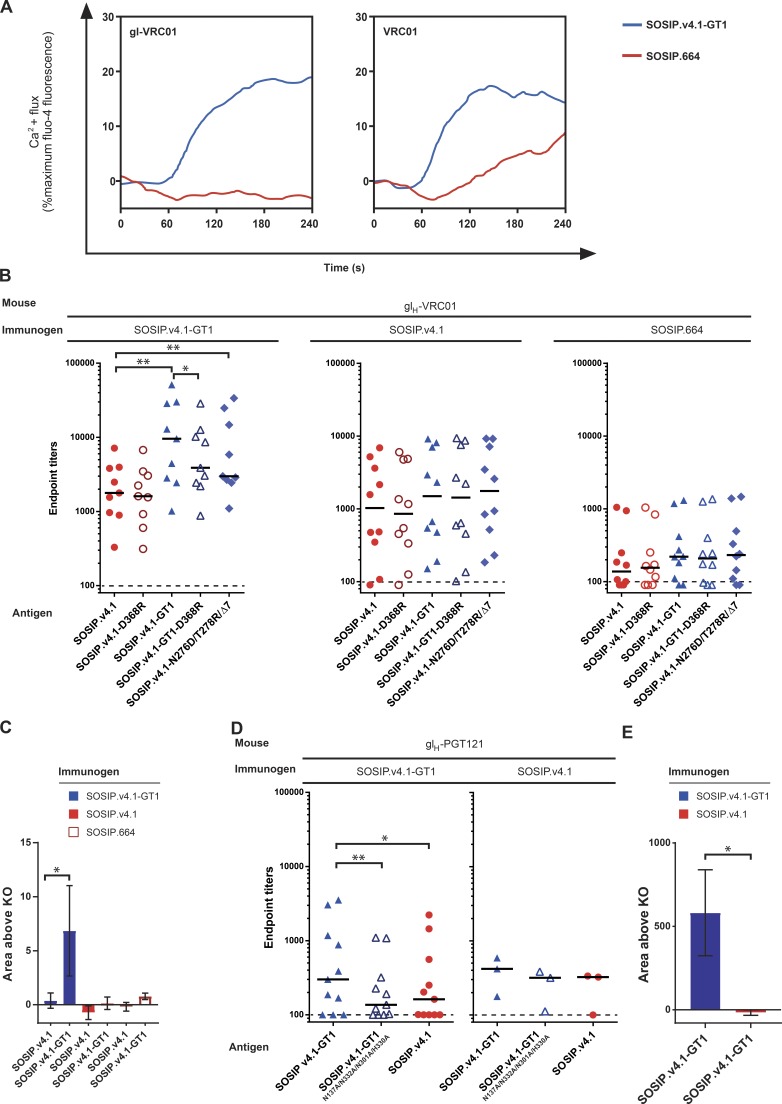
**BG505 SOSIP.v4.1-GT1 initiates antibody responses in knock-in mice expressing the predicted germlines of VRC01 and PGT121 bNAbs.** (A) Calcium flux in B cells expressing either gl-VRC01 (left) or VRC01 (right) as a B cell receptor, stimulated with the indicated trimers at a 1 µM final concentration. In this and subsequent panels, all of the trimers used as immunogens and ELISA antigens were of the BG505 genotype. (B) Endpoint antibody binding titers in sera from gl_H_-VRC01 mice immunized thrice with SOSIP.v4.1-GT1 (left), SOSIP.v4.1 (center), or SOSIP.664 (right) trimers, measured against the indicated His-tagged BG505 trimer variants by ELISA. The median titers are indicated by the black lines. Statistically significant differences are indicated by asterisks (*, P ≤ 0.05; **, P ≤ 0.01; Wilcoxon matched-pairs signed rank test). The ELISA curves can be found in Fig. S5 A. (C) Antibody specificity determinations. For each mouse, the area under the curve for a given gl-VRC01 epitope knockout trimer (SOSIP.v4.1-D368R or SOSIP.v4.1-GT1-D368R) was subtracted from the area under the curve obtained with the corresponding unmodified trimer (i.e., SOSIP.v4.1 or SOSIP.v4.1-GT1). The resulting “area above knockout (KO)” values are plotted as bars. The ELISA curves used for the area-under-the-curve analyses can be found in Fig. S5 A. The method has been described before. The mean and SEM are indicated. Statistically significant differences are indicated by asterisks (*, P ≤ 0.05; Wilcoxon matched-pairs signed rank test). (D) Endpoint antibody binding titers in sera from gl_H_-PGT121 mice immunized thrice with either SOSIP.v4.1-GT1 (left) or SOSIP.v4.1 (right) trimers, measured against the indicated His-tagged BG505 trimer variants by ELISA. The median titers are indicated by the black lines. Statistically significant differences are indicated by asterisks (*, P ≤ 0.05; **, P ≤ 0.01; Wilcoxon matched-pairs signed rank test). The ELISA curves can be found in Fig. S5 B. (E) Antibody specificity determinations. For each mouse, the area under the curve for the PGT121 epitope knockout trimer (SOSIP.v4.1-GT1-N137A/N332A/N301A/H330A) was subtracted from the area under the curve obtained with the unmodified trimer (i.e., SOSIP.v4.1-GT1). The resulting area above KO values are plotted as bars. The ELISA curves used for the area-under-the-curve analyses can be found in Fig. S5 B. The mean and SEM are indicated. Statistically significant differences are indicated by asterisks (*, P ≤ 0.05; Wilcoxon matched-pairs signed rank test).

We next evaluated whether the BG505 SOSIP.664 and GT1 trimers could activate B cell lines expressing the gl version of VRC01. The GT1 trimers did indeed activate gl-VRC01 B cells, whereas the parental SOSIP.664 trimers were ineffective (Fig. 5 A). Although both trimers activated B cells expressing the mature VRC01, the GT1 variant was better (Fig. 5 A). Thus, the improved gl-VRC01 binding properties of the GT1 trimer translate into superior activation of B cells carrying a gl-VRC01 BCR.

To assess whether the above in vitro observation is predictive of what happens when a GT1 trimer encounters a naive VRC01-class B cell in vivo, we immunized mice expressing the inferred gl *IgH* gene of VRC01 (i.e., gl_H_-VRC01 knock-in mice; Jardine et al., 2015). One group of mice received GT1 trimers, and for comparison, two others were given SOSIP.664 or SOSIP.v4.1 trimers. The outcome of the experiment was determined by measuring binding antibody responses in sera, using a capture ELISA based on His-tagged versions of the immunogen trimers and mutants thereof in which relevant epitopes were inactivated. Binding antibody responses were significantly higher in the mice immunized with GT1 trimers when measured against the GT1 trimer than SOSIP.v4.1 (P = 0.003) or the GT1-D368R mutant (P = 0.01). Thus, a substantial fraction of the antibody response was against epitopes that are better displayed on the GT1 trimers and that involve residue D368 in the CD4bs (Fig. 5, B [left] and C; and Fig. S5 A). We also used a mutant trimer SOSIP.v4.1-N276D/T278R/Δ7 that included two substitutions in loop D (N276D and T278R) to enhance accessibility of the CD4bs and a seven-residue deletion in V2. These changes are also present in the GT1 trimer and allowed moderate binding to gl-VRC01 (Fig. S1 C and Table S5). The binding responses in the GT1 trimer-immunized mice were significantly higher when measured against this mutant compared with SOSIP.v4.1 (P = 0.003), which is further evidence for the elicitation of antibodies that recognize the CD4bs (Fig. 5 B, left; and Fig. S5 A).

The antibody responses in mice immunized with the SOSIP.v4.1 or SOSIP.664 control trimers were substantially lower than in the GT1 trimer-immunized animals, irrespective of the trimer used in the detection ELISA (Fig. 5 B). The reduced response was particularly striking for the SOSIP.664 immunized animals. In these two groups of control animals, the binding antibody responses were not statistically significantly affected by GT1 substitutions (GT1 vs. SOSIP.v4.1), by reduction of VRC01 contacts (GT1 vs. GT1-D368R), or by enhanced exposure of the CD4bs (SOSIP.v4.1 vs. SOSIP.v4.1-N276D/T278R/Δ7; Fig. 5, B [middle and right] and C). The implication is that the antibodies elicited in these mice predominantly recognize epitopes that are not specific for the CD4bs (i.e., irrelevant off-target responses), probably stemming from the remaining B cells that express mouse BCRs (Jardine et al., 2015).

### In vivo activation of B cells expressing gl-PGT121

To determine whether GT1 trimers could trigger gl antibody responses to epitopes outside the CD4bs, we immunized knock-in mice expressing the predicted gl *IgH* gene of the N332/V3-base directed bNAb PGT121 (i.e., gl_H_-PGT121 knock-in mice; Escolano et al., 2016). These mice were given either the GT1 trimer or, for comparison, SOSIP.v4.1. The antibody responses among the 11 GT1 trimer-immunized mice were very variable, but were significantly higher when measured against the GT1 trimer than against a mutant trimer containing four substitutions that knock out the PGT121 epitope (GT1-N137A/N332A/N301A/H330A; P = 0.007). They were also higher when measured against GT1 trimers than SOSIP.v4.1 (P = 0.01). The implication is that, in at least some of the mice, the antibody responses to the GT1 trimer are targeting the PGT121 epitope (Fig. 5, D [left] and E; and Fig. S5 B). These results are striking in light of the undetectable binding of the GT1 trimer to gl-PGT121 in ELISA and SPR assays (Figs. S1 B and S2 and Table S7), but in agreement with a previous study showing that PGT121 responses could be initiated in vivo with a protein that had no measureable affinity to gl-PGT121 (Escolano et al., 2016; Steichen et al., 2016). The SOSIP.v4.1 control trimer induced a low level of trimer binding antibodies in the gl_H_-PGT121 knock-in mice, but there was no difference in their recognition of the GT1 trimer, the SOSIP.v4.1 trimer, or the GT1-N137A/N332A/N301A/H330A (Fig. 5, D and E) designed to knock out PGT121 binding (Fig. S1 D). It is, therefore, likely that these antibodies are not specific for the PGT121 epitope but are off-target responses.

We conclude that the BG505 SOSIP.v4.1-GT1 trimer can activate B cells expressing gl versions of two different bNAbs to two different epitope clusters under in vivo conditions.

## Discussion

The concept of targeting gl antibody precursors is now acknowledged as an important strategy for HIV-1 Env vaccines that are intended to induce bNAbs in humans (Scheid et al., 2009; Xiao et al., 2009b; Escolano et al., 2017; Medina-Ramírez et al., 2017; Sanders and Moore, 2017; Stamatatos et al., 2017; Verkoczy et al., 2017). Thus, Env immunogens must be designed to engage and activate naive B cells expressing gl antibodies that have the potential to evolve into a bNAb. Subsequent boosting by a different or modified immunogen may then help drive the somatic hypermutation events required to evolve bNAbs. How then can Env immunogens be designed to target gl versions of bNAbs? Env proteins tend to react poorly with gl-bNAbs, as they are based on sequences that have been shaped by the antibody response to HIV-1 infection and the particular virus that initiated the response is often not known (Scheid et al., 2009; Xiao et al., 2009a,b; Mouquet et al., 2010; Zhou et al., 2010; Haynes et al., 2012; Klein et al., 2013b; Liao et al., 2013; Doria-Rose et al., 2014; Lynch et al., 2015). Accordingly, Env proteins must be redesigned to create immunogens that can bind gl-bNAbs with high affinity in vitro and, by extension, activate the analogous naive B cells in vivo.

Our approach was based on modifying native-like recombinant SOSIP trimers based on the BG505 sequence. The BG505 SOSIP.664 trimer and its more stable SOSIP.v4.1 derivative bind multiple bNAbs in vitro and elicit autologous Tier-2 NAbs in animals (de Taeye et al., 2015; Sanders et al., 2015; Klasse et al., 2016). They do not, however, induce bNAb responses. Moreover, although the BG505 SOSIP.664 trimer binds to gl precursors of the PG9/16, CH01 and 3BC315 bNAbs in vitro, it fails to react with several others, including all those tested from the VRC01 class (Sliepen et al., 2015).

Here, we describe the reengineering of the BG505 SOSIP.664 trimer to increase affinity for gl precursors of multiple bNAb lineages. The key elements of the design strategy involve removing steric clashes that hinder gl binding and creating favorable new antibody-antigen contacts that promote selection of the appropriate gl-bNAb. The 3.2-Å structure of the resulting GT1 trimer, particularly when compared with the SOSIP.664 structure, permits a mechanistic dissection of how it engages gl-bNAbs. The GT1 trimer is fully native-like; has biochemical, biophysical, and expression properties comparable with its SOSIP.664 and SOSIP.v4.1 precursors; and can be purified to structural homogeneity by bNAb affinity chromatography. These characteristics offer a practical path to producing the GT1 trimer as an immunogen for further testing in animals and, perhaps, eventually in humans.

On ELISA, the GT1 trimer bound two- to fivefold more strongly to three gl-bNAbs against trimer-apex epitopes (gl-PG9, gl-PG16, and gl-CH01) compared with its precursors. The GT1 trimer also gained the ability to bind strongly to CD4bs gl-bNAbs gl-VRC01, gl-NIH45-46, and gl-PGV19, moderately to gl-12A12, and weakly to gl-CH31 and gl-CH103 (Fig. 2, A and B; and Fig. S1 B). We tested the immunogenicity of the GT1 and control trimers in knock-in mice expressing the gl precursors for two different bNAbs: VRC01 to the CD4bs and PGT121 to the N332/V3-base cluster. Antibodies with characteristics consistent with the respective gl-bNAbs were induced in both models in response to the GT1 trimer immunogen, as judged by their ELISA reactivity with the same trimer and mutants with sequence changes affecting the target epitope. In contrast, the SOSIP.v4.1 or SOSIP.664 control trimers did not induce antibodies with these properties (Fig. 5, B–E; and Fig. S5).

The GT1 trimer was not designed to activate gl-PGT121 B cells and did not bind to gl-PGT121 in ELISA and SPR experiments. Nevertheless, the GT1 trimer, but not the parental trimer, initiated an epitope-specific response in vivo. Thus, (the lack of) binding by SPR does not necessarily predict the outcome of in vivo experiments (Escolano et al., 2016). We do not know how the GT1 trimer activates gl-PGT121 B cells, but the changes made in GT1 might have enhanced access to the PGT121 epitope in vivo.

Of note is that in SPR analyses, GT1 resembles the unmodified SOSIP.v4.1 trimer by binding with high affinity to mature CD4bs bNAbs, such as VRC01, and not reacting with CD4bs antibodies F105 and b6 that are non-NAbs against Tier-2 viruses. The retention of trimerization-induced constraints on the CD4bs epitopes suggests that the GT1 trimer may have the selectivity to induce desired lineages (e.g., for VRC01-like bNAbs) without activating “off-target” lineages (e.g., non-NAbs such as F105 and b6) in vivo. This property could be highly advantageous because the angle of approach to the trimer of several bNAb lineages analyzed to date appears to be established at the gl stage, with only relatively minor changes during affinity maturation (Jardine et al., 2013; Zhou et al., 2013; Fera et al., 2014; Garces et al., 2015; Scharf et al., 2016). Accordingly, off-target lineages might never yield bNAbs. In summary, the conformational selectivity offered by SOSIP.v4.1-GT1 trimers may help to determine the appropriate selection and development of CD4bs bNAb lineages.

Two other gl-targeting Env immunogen designs, the 426c and eOD proteins, have been specifically constructed to induce precursors of the VRC01-family of CD4bs bNAbs (Stamatatos et al., 2017). However, as the CD4bs regions of the 426c and eOD proteins are not constrained by trimerization, they may also present non-NAb epitopes. It is not yet known whether the presence of such “off-target” epitopes matters from the perspective of inducing VRC01 gl-bNAbs that can then be shaped by boosting with a second immunogen, such an Env trimer.

As noted, the GT1 trimer was engineered to bind inferred gl-bNAbs targeting several epitope clusters, not just a single one (Jardine et al., 2013, 2015; Dosenovic et al., 2015; Escolano et al., 2016; Sok et al., 2016; Steichen et al., 2016), and it has the appropriate antigenicity properties in vitro. These design features may broaden the human repertoire of gl-bNAb precursors that can be targeted and increase the probability that at least one family of gl-bNAb B cells will be activated in vivo. Could the current GT1 trimers be further modified to present more gl-bNAb epitopes, such as those at the gp120-gp41 interface? Could a “universal gl-targeting trimer” be created? Or would it be better to design a suite of different trimers that individually target a specific gl-bNAb cluster? These questions can only be addressed experimentally. For example, removing multiple glycans that clash with a variety of gl-bNAbs may have adverse structural consequences and alter glycan processing. Furthermore, improving the targeting of one epitope cluster may also adversely affect a neighboring or even a distant one.

Although these are encouraging initial indicators of appropriate immunogenicity, the limitations of knock-in mouse models must be recognized. For example, in the knock-in mice, a far higher proportion of B cells express the gl-bNAb receptor than would be the case in humans. Thus, to achieve a similar response in humans, it may be necessary to further increase an Env immunogen’s affinity for the gl antibodies being targeted while preventing potential competition from unwanted binders (Escolano et al., 2017; Medina-Ramírez et al., 2017; Sanders and Moore, 2017; Stamatatos et al., 2017).

This new gl-targeting trimer is suitable for further evaluation as an immunogen to gauge its abilities to induce gl-bNAb lineages and the specificity with which it does so. In all likelihood, it will need then to be combined with carefully designed boosting immunogens to ensure that a bNAb response is appropriately shaped and productively matured.

## Materials and methods

### Construction of a BG505.T332N-LAI chimeric molecular clone

The molecular clone of LAI was used as the backbone (Peden et al., 1991). This clone contains a unique Sal1 restriction site 434 nucleotides upstream of the *env* start codon and a unique BamH1 site at the codons specifying amino acids G751 and S752 in LAI gp160 (HxB2). A DNA fragment was synthesized containing the LAI sequences between the Sal1 site and the *env* start codon, followed by the BG505.T332N *env* sequences up to the BamH1 site (Genscript) and cloned into the LAI molecular clone backbone using Sal1 and BamH1. The resulting molecular clone encodes the complete BG505.T332N gp160 sequence, except for the C-terminal 106 amino acids of the cytoplasmic tail, which are derived from LAI gp160. The resulting virus was able to infect TZM-bl cells and replicate in PBMCs.

### Neutralization assays

The virus neutralization activities of antibodies targeting the trimer apex were assessed using the TZM-bl cell assay as described elsewhere (Sanders et al., 2015). The PG9, PG16, and PGT145 bNAbs were tested at single concentrations of 5 µg/ml (mature versions) and 50 µg/ml (gl versions). All experiments were performed in triplicate. The assay endpoint (percentage neutralization) was calculated relative to the extent of HIV-1 infection measured in the absence of antibody.

### Trimer expression and purification

Env proteins were expressed by transient transfection of adherent HEK293T cells (incubated for 48 h) or suspension FreeStyle 293F cells (Invitrogen; incubated for 6 d), as described previously (Julien et al., 2013; Sanders et al., 2013). Env proteins were purified from culture supernatants by PGT145-affinity chromatography (de Taeye et al., 2015). Trimer cleavage and purity was assessed using SDS-PAGE and BN-PAGE analyses (Sanders et al., 2013).

### Env trimer design and mutagenesis

To create the BG505 SOSIP.v4.1-GT1 trimer, 17 individual point substitutions and a seven amino acid deletion were introduced into the BG505 SOSIP.664 construct (Fig. 1, A and B) using the QuikChange site-directed mutagenesis kit (Agilent Technologies). Specific epitope knockout substitutions (D368R for VRC01 and N137A/N332A/N301A/H330A for PGT121), as well as substitutions that removed the N276 glycan and seven amino acids from V2 (N276D/T278R/Δ7), were introduced using the same method. His-tagged or D7324-tagged versions of the same or similar trimers were also produced (Sanders et al., 2013; de Taeye et al., 2015). His- and D7324-tagged trimers were used in ELISA, His-tagged trimers in B cell activation assays, and SPR (see below), whereas NS-EM, DSC, and crystallography studies were performed with nontagged trimers. The presence or absence of these epitope tags does not influence the structure of the trimer (Sanders et al., 2013). The purities of trimers were assessed using BN- and SDS-PAGE followed by staining with Coomassie blue as described previously (Sanders et al., 2013). The biochemical and biophysical assays for Env trimer characterization have all been published elsewhere (de Taeye et al., 2015).

### ELISA for trimer antigenicity

We adapted an ELISA protocol as previously described (Derking et al., 2015). In brief, His-tagged trimers, either pure (3.5 µg/ml in TBS buffer) or in unpurified HEK293T cell culture supernatant (His- and D7324-tagged; supplemental information), were immobilized (100 µl/well) for 2 h on 96-well Ni-NTA ELISA plates (QIAGEN) or 96-well ELISA plates coated overnight with D7324 antibody (Aalto Bioreagents). After washing away excess protein with TBS, the wells were blocked for 30 min with casein/TBS (37532; Thermo Fisher Scientific). Serial dilutions of each antibody were prepared in casein/TBS at a starting concentration of 1 µg/ml and added to the plate (100 µl/well; for lower affinity antibodies, the starting concentration was 50 µg/ml). The dilution factor for all antibodies was 1:3 except for gl-CH103, which was 1:2. Excess antibody was washed away after 2 h and antihuman HRP-conjugated antibody (diluted in casein/TBS 1:3,000) added for 45 min before binding was quantified. All steps were performed at room temperature.

### Analysis of total N-glycan profile by hydrophilic interaction liquid chromatography–ultraperformance liquid chromatography

*N*-linked glycans were enzymatically released by in-gel PNGase F digestion from trimers resolved by nonreducing SDS-PAGE. The released glycans were fluorescently labeled with 2-aminobenzoic acid and analyzed as previously described (Pritchard et al., 2015).

### B cell activation assays

DG75 B cells were transfected by electroporation with a plasmid expressing the mature VRC01 IgG BCR. After 24 h, the cells were loaded with Fluo-4 direct Ca^2+^ indicator dye and then stained with an anti-IgG antibody labeled with BV421 to identify transduced cells. Baseline Fluo-4 fluorescence was measured for 30 s, after which the indicated recombinant Env proteins were added to a final concentration of 1 µM. Changes in Fluo-4 fluorescence were monitored for an additional 210 s. Ionomycin was then added to a final concentration of 6.5 nM for an additional 60 s of fluorescence monitoring. Maximum Fluo-4 fluorescence (Max_FL_) was established by averaging the fluorescence changes recorded during the last 10 s of monitoring. The percentage of maximum Fluo-4 fluorescence at each time point, *t*, was determined using the formula (fluorescence at *t* − Min_FL_)/(Max_FL_ − Min_FL_) × 100. This analysis was performed on both transfected and untransfected cells simultaneously. The background Fluo-4 fluorescence signal from the BCR-negative cells was subtracted from that of the BCR-positive population at each time point. The same analysis was performed for DG75 B cells stably transduced to express the gl-VRC01 BCR (McGuire et al., 2016).

### DSC

DSC was used to determine the thermostability of purified trimers, as described previously (de Taeye et al., 2015; Pugach et al., 2015).

### SPR

SPR was performed as previously described with immobilized His-tagged trimers and antibodies (IgG) as the analytes; binding parameters were derived by applying a bivalent model (Yasmeen et al., 2014). The bivalent model dissects the initial monovalent from the subsequent bivalent binding, as previously validated by comparing IgG with Fabs and trimers at different densities (Yasmeen et al., 2014). Here we used the standard level of trimer immobilization, *R_L_* = 500 RU, which falls in the range of trimer densities on virions that have been estimated and typically gives a low degree of bivalency (Klein and Bjorkman, 2010; Yasmeen et al., 2014). Overall, the bivalent component reproducibly represented a minority of the binding events. Here, we converted the units of the bivalent constants *k_on2_* and *K_d2_* from (1/RUs) and (RU) to (1/Ms) and (nM), by taking into account the reaction volume on the SPR chip and the specific signal per mass unit of analyte. These considerations give the formula 1 [1/(RUs)] ≈100 **·** M_A_ [1/(Ms)], where M_A_ is the molar mass of the analyte, as described previously (Karlsson et al., 1995). Although this conversion conveniently confers the same dimension to the mono- and bivalent on-rate constants, it should be born in mind that the unoccupied paratopes do not diffuse freely, and their local concentrations in relation to the epitope-presenting trimers immobilized to dextran remain unknown. Therefore, the constants for bi- and monovalent binding are not directly comparable despite the conversion. Nevertheless, the *K_d1_* values were lower than the *K_d2_* values in all cases but one: BG505 SOSIP.664 and mature PG16 antibody, for which the two constants were similar (52 vs. 30 nM). A strong bivalent contribution to the binding would manifest itself as substantially lower *K_d2_* than *K_d1_* values. The low degree of bivalency was also evident from comparisons that do not depend on the above conversion: component analyses of each binding cycle modeled bivalently and a comparison of the T values, indicating significance of the fitted parameter, for the *k_on1_* and *k_on2_* values (the off-rate constants being less amenable to comparison because *k_off1_* was frequently below detection). The T values for *k_on1_* were consistently >10, with a minimum of 63; the T value for *k_on2_* was <10 in ∼25% of the cases, with a minimum of 3.1. Such weak bivalency is to be expected at a trimer density that is in the range of what occurs on virion surfaces (Klein and Bjorkman, 2010). We therefore conclude that the kinetic and stoichiometric measurements that we obtained for the monovalent paratope-epitope interaction were largely unaffected by the highly limited propensity for bivalent interaction, in line with previous comparisons of Fab with IgG binding and the Langmuir with bivalent modeling (Yasmeen et al., 2014).

An advantage of using IgG rather than Fabs, apart from obviating the need for Fab production and purification of all antibodies, is a stronger signal through the threefold greater mass, allowing detection of weak gl-bNAb binding. Furthermore, the use of IgG incorporates unusual allosteric effects transmitted from the Fc portion to the paratope (Crespillo et al., 2014), manifestations of asymmetries in the IgG molecules (Saphire et al., 2002), and reduction in epitope accessibility on immobilized trimers by the bulk of the IgG molecule (Labrijn et al., 2003).

### NS-EM

NS-EM assessed Env trimer morphology following previously described procedures (Sanders et al., 2013; de Taeye et al., 2015; Pugach et al., 2015).

### Mice and immunizations

The gl_H_-PGT121 mice (carrying the *Ig V*[*D*]*J* genes encoding the gl *IgH*) were produced by gene targeting Albino B6 (B6 [Cg]-*Tyrc-2J*/J) embryonic stem cells. The amino acid sequence of the heavy chain of gl-PGT121 (Table S1) was previously described (Escolano et al., 2016). The constant regions of *IgH* as well as the *IgL* diversity remain of mouse origin. The targeting vectors for *IgH* contained homologous regions flanking mouse *D4-1* and *J4*. Recombination results in the deletion of the endogenous *D4-1* and *Js*, thereby minimizing rearrangement of the locus (Pelanda et al., 1997; Shih et al., 2002). The gl_H_-VRC01 knock-in mice have been described elsewhere (Jardine et al., 2015).

Two and three independent experiments were performed using the gl_H_-VRC01 and gl_H_-PGT121 mice, respectively. Mice were immunized three times every 2–4 wk intraperitoneally with 10 µg protein in Ribi adjuvant (Sigma-Aldrich). Serum samples were collected 2 wk after the third immunization. All animal procedures were performed in accordance to protocols approved by The Scripps Research Institute (VRC01 mice) or The Rockefeller University (all other mice) Institutional Animal Care and Use Committee.

### ELISA for antitrimer antibodies in mouse sera

ELISAs to measure serum responses to the BG505 SOSIP variants were adapted from elsewhere (Yasmeen et al., 2014; Derking et al., 2015; Dosenovic et al., 2015). In brief, His-tagged antigen was captured by using Ni-NTA ELISA plates (QIAGEN) or, alternatively, with an anti-His_6_-tag antibody (Abcam). Plates coated overnight with anti-His_6_-tag antibody were washed six times (PBS with 0.05% Tween 20 [Sigma-Aldrich] or TBS) and blocked in blocking buffer (1× PBS with 1% milk) for 1 h at room temperature. Immediately after blocking (no blocking for Ni-NTA plates), His-tagged GT1, SOSIP.v4.1, or SOSIP.664 trimer (or mutants thereof) was added at 3.5 µg/ml in TBS (or 2 µg/ml diluted in PBS with 1% FBS and 0.2% Tween-20 for antigens captured with the anti-His_6_ antibody) to all the wells and incubated at room temperature for 1 or 2 h. Plates were then washed and blocked for 1 h at room temperature. After blocking, serum samples were added in PBS with 1% FBS and 0.2% Tween-20 (for antigens captured with the anti-His_6_ antibody) or 2% skim milk in TBS supplemented with 20% sheep serum (Biotrading) for the Ni-NTA plates and incubated for 2 h at 37°C. Sera were added at 1:100 starting dilution. Seven additional threefold serial dilutions were made. Plates were washed and incubated for 1.5 h at 37°C with an HRP-antimouse IgG antibody (The Jackson Laboratory; in PBS with 0.05% Tween-20 or 2% skim milk in TBS) at a 1:5,000 dilution. Plates were developed by addition of the HRP substrate, ABTS (Thermo Fisher Scientific), and absorbance was measured with an ELISA microplate reader (at 405 nm in a FluoStar Omega, BMG Labtech, or at 450 nm in a Spectrostar nano, BMG Labtech).

### Expression and purification of proteins for x-ray crystallographic studies

The 9H+109L and 35O22 Fabs were produced by transient transfection of FreeStyle 293F cells and purified by affinity chromatography using CaptureSelect LC lambda (Thermo Fisher Scientific), followed by cation exchange and size exclusion chromatography (SEC) on a Superdex 200 16/60 column (GE Healthcare). The BG505 SOSIP.v4.1-GT1-N137A construct was cloned into a phMCV3 vector, expressed in FreeStyle 293S cells (incubated for 6 d) and trimers purified by 2G12-affinity chromatography followed by SEC. The purities of trimers and Fabs were assessed using SDS-PAGE, followed by staining with Coomassie blue as described previously (Sanders et al., 2013).

### Crystallization and data collection

Multiple combinations of Fabs and BG505 SOSIP.v4.1-GT1 trimers (including the substitution N137A) were assessed for complex formation in crystallization trials. Generally, Fabs and trimers were mixed in a 3.2:1 molar ratio. To increase the homogeneity of trimer–ligand complexes, a deglycosylation procedure was performed as described previously (Garces et al., 2015), followed by further SEC purification.

To facilitate crystal packing, the GT1 trimer was complexed with Fab 35O22 and Fabs from the PGT121 family, including 9H+109L and 3H+109L (Pancera et al., 2014; Garces et al., 2015). Samples of SEC-purified ternary complexes were concentrated to ∼12 mg/ml and screened against 480 crystallization conditions at 4°C using the robotic Rigaku CrystalMation system at TSRI. The most successful combination, SOSIP.v4.1-GT1 in complex with Fabs 9H+109L and 35O22, was initially crystallized in JCSG Core II Suite (QIAGEN) condition E12 (0.2 M sodium chloride, 0.1 M sodium acetate, 40% PEG 300, pH 4.5) and, after further optimization, crystals for data collection were grown in 0.2 M sodium chloride, 34% (vol/vol) PEG 300, and 0.1 M sodium acetate, pH 4.5. Crystals were flash cooled in liquid nitrogen using the well solution as cryoprotectant, and data were collected at Advanced Photon Source beamline 23-ID-D. Despite the best crystal producing a few strong reflections beyond 3.0-Å resolution, the final dataset was processed with HKL-2000 (Otwinowski and Minor, 1997) to 3.2 Å with an overall *R*_sym_ of 0.11 and 100% completeness in space group P6_3_ with unit cell parameters *a* = *b* = 128.0 Å, *c* = 316.1 Å (Table S8; Weiss and Hilgenfeld, 1997).

### Structure determination and refinement

The structure was solved by molecular replacement with Phaser (Adams et al., 2010) using the BG505 SOSIP.664 structure in complex with 3H+109L and 35O22 (PDB accession no. 5CEZ) as the search model. Model building was performed using Coot (Emsley and Cowtan, 2004) and refinement with phenix.refine (Adams et al., 2010) using reference model restraints calculated from structures of BG505 SOSIP.664 with 3H+109L and 35O22 (PDB accession no. 5CEZ) and 35O22 (PDB accession no. 4TOY). The final *R*_cryst_ and *R*_free_ values were 27.2% and 28.5% (Table S8). The Fab residues were numbered according to Kabat et al. (1991) and gp120 and gp41 residues using HxB2 numbering. Ramachandran statistics were calculated using MolProbity (Chen et al., 2010).

### Data processing and statistical analysis

The Geneious 9.0.4 and MacVector 14.0.3 programs were used for sequence analysis. Flow cytometry data were processed using FlowJo 10.0.7. GraphPad Prism 6.0f was used for data and statistical analysis by one-way ANOVA and the Tukey multiple-comparison test. Data were considered statistically significant at P ≤ 0.05.

### Accession numbers

Env sequence data for isolates H19463, H18969, and H19792 are available from GenBank under accession nos. JF910186, EU744055, and JF910175. The coordinates and structure factors of the BG505 SOSIP.v4.1-GT1 trimer crystal structure with Fabs 35O22 and 9H+109L have been deposited in the PDB under accession no. 5W6D.

### Online supplemental material

Fig. S1 shows representative ELISA binding curves to multiple BG505 SOSIP trimer variants using a panel of mature bNAbs and gl-bNAbs. Figs. S2 and S3 show SPR analysis of binding of bNAbs, gl-bNAbs, and non-NAbs to three versions of BG505 SOSIP trimers. Fig. S4 shows structural analysis of bNAb precursor engagement to BG505 SOSIP.v4.1-GT1-N137A. Fig. S5 shows representative ELISA binding curves to multiple BG505 SOSIP variants using sera derived from immunization of gl_H_-VRC01 and gl_H_-PGT121 knock-in mice. Table S1 lists the heavy- and light-chain sequences of the gl-bNAbs used in this study. Table S2 shows the analysis of gl-bNAb neutralization sensitivity of a panel of viruses. Tables S3, S4, and S5 show the relative binding capacity of a panel of three gl-bNAbs and two bNAbs to BG505 SOSIP trimer variants. Table S6 shows the percentage of Man_5-9_GlcNAc_2_ glycans (M5-M9) in three BG505 SOSIP trimer variants. Table S7 shows the SPR analysis of a panel of mature bNAbs and gl-bNAbs to BG505 SOSIP trimer variants. Table S8 shows the x-ray data collection and refinement statistics.

## Supplementary Material

Supplemental Materials (PDF)

Table S2 (Excel file)

Table S7 (Excel file)
