# Supplementary material for: Design and crystal structure of a native-like HIV-1 envelope trimer that engages multiple broadly neutralizing antibody precursors in vivo
Source: J Exp Med. 2017 Sep 4;214(9):2573–90. doi: 10.1084/jem.20161160 (PMC5584115; doi:10.1084/jem.20161160)
Supplement: Supplemental Materials (PDF) [file JEM_20161160_sm.pdf]

The Journal of Experimental Medicine

SUPPLEMENTAL MATERIAL

Medina-Ramírez et al., https://doi.org/10.1084/jem.20161160

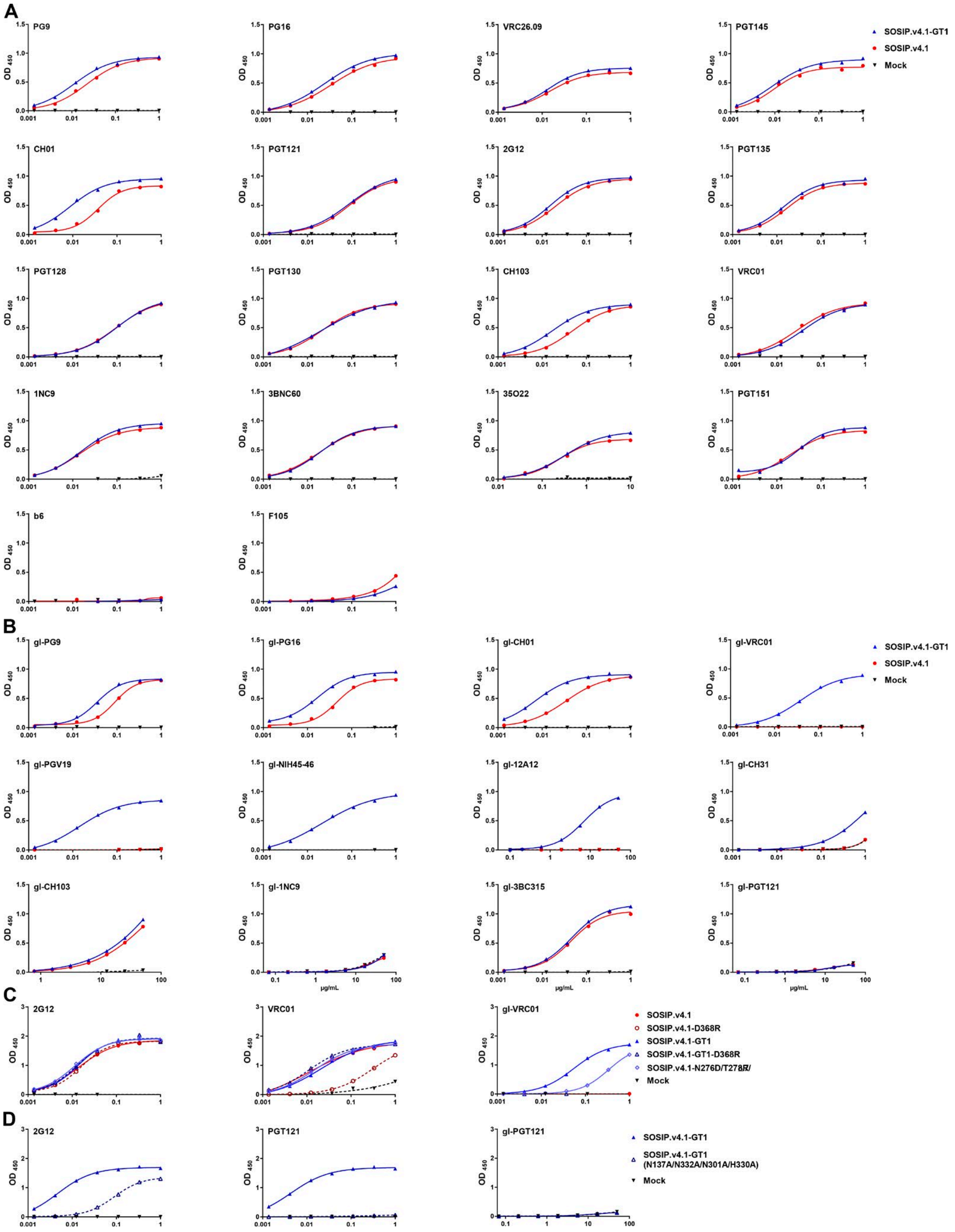

Figure S1. **Antigenicity of BG505 SOSIP.v4.1 and SOSIP.v4.1-GT1 trimers and analyses of epitope knockout trimer mutants by ELISA (related to Fig. 2 A; and Fig. 5, B–E).** The representative ELISA binding curves were derived using a panel of (A) mature bNAbs and (B) gl-bNAbs. The dilution factor for all antibodies was 1:3 except for gl-CH103, for which the dilution factor used was 1:2. (C) Binding of 2G12 (left), VRC01 (middle), and gl-VRC01 (right) to the trimer variants used for the analyses in Fig. 5. The overlapping 2G12 titration curves indicate that equivalent amounts of the various trimers (3.5 µg/ml) were captured onto the ELISA wells. Binding of the mature VRC01 bNAb was reduced ∼10-fold when the D368R substitution was introduced into the SOSIP.v4.1 trimer but was not affected when the same D368R substitution was made in the SOSIP.v4.1-GT1 context. A possible explanation is that the loss of the antibody-trimer contact caused by the D368R change is compensated by other substitutions in and around the CD4bs of the GT1 trimer that are not present in SOSIP.v4.1. The gl-VRC01 bNAb precursor did not bind the SOSIP.v4.1 trimer but did bind the engineered GT1 variant. However, gl-VRC01 binding was no longer detectable when the D368R change was introduced into the GT1 construct. The gl-VRC01 antibody also bound the SOSIP.v4.1-N276D/T278R/Δ7 trimer mutant that lacked the N276 glycan and 7 amino acids in V2, albeit less well than the GT1 trimer. (D) Binding of 2G12 (left), PGT121 (middle), and gl-PGT121 (right) to the SOSIP.v4.1-GT1 trimer and the SOSIP.v4.1-GT1-N137A/N332A/N301A/H330A PGT121 epitope knockout mutant. Although the four substitutions cause a partial reduction in 2G12 reactivity, PGT121 binding to the GT1 trimer is completely eliminated. The gl-PGT121 precursor does not bind to either the GT1 trimer or the quadruple mutant.

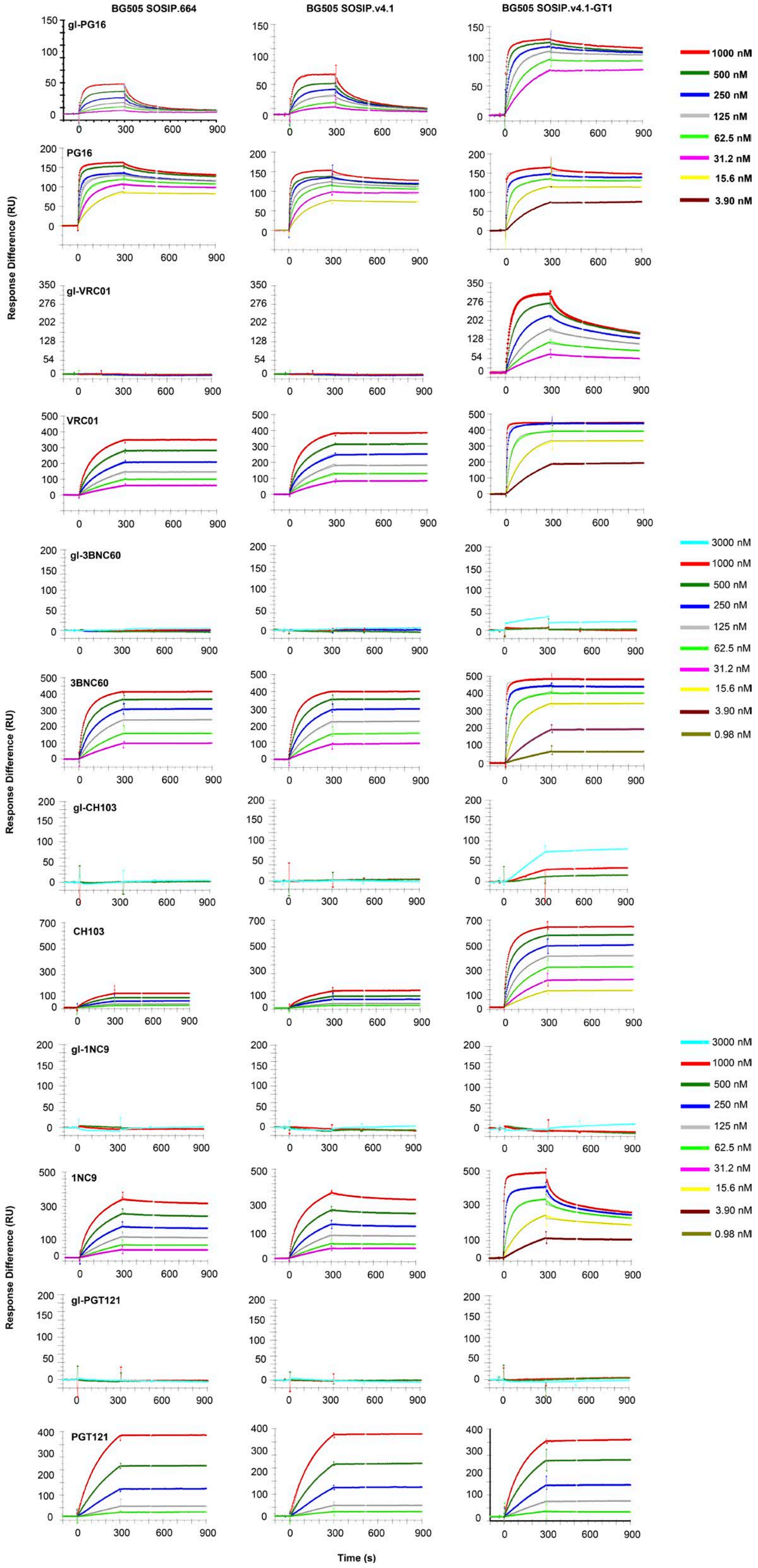

Figure S2. **SPR analysis of binding of a panel of six bNAbs and their corresponding germline versions to three versions of BG505 SOSIP trimers (related to Fig. 2 B and Table S7).** The antibodies tested bind to a variety of epitopes (apex, CD4bs, and N332/V3). The sensorgrams show the response (RU) over time (s). The association phase was 300 s and, dissociation was followed over 900 s. Curves for concentration ranges (see inset) are shown in color.

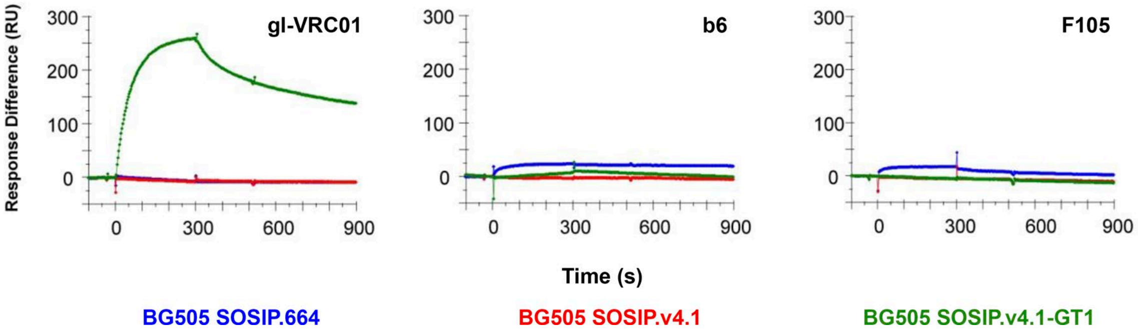

Figure S3. **Antibody binding to Env trimers (related to Fig. 2 B).** SPR analysis of the binding of gl-VRC01, b6, and F105 to three versions of BG505 SOSIP trimers. The sensorgrams show the response (RU) over time (s). The association phase was 300 s, and dissociation was followed over 900 s. The colored curves show the responses obtained at a concentration of 500 nM of each antibody.

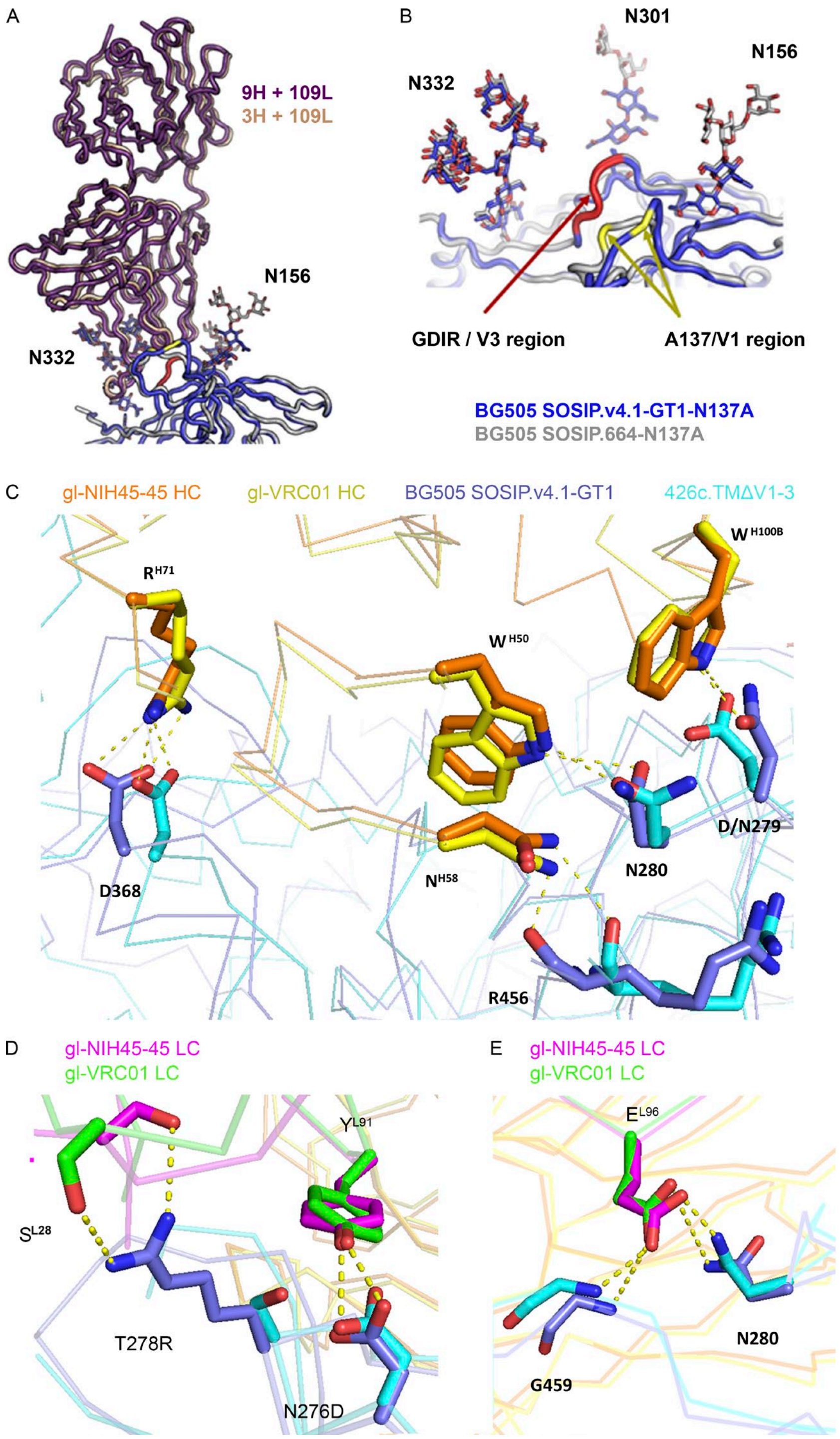

Figure S4. **Analysis of the 3H+109L and 9H+109L epitopes on the BG505 Env trimer and comparison of signature VRC01-class contacts in gl-bNAb Env complexes (related to Figs. 3 and 4).** (A) Superimposition of the crystal structure of Fab 9H+109L-BG505 SOSIP.v4.1-GT1-N137A complex with the Fab 3H+109L-BG505 SOSIP.664-N137A complex. The antibodies (putative heavy chain precursors of the PGT121 family) and Env trimer are depicted as colored tubes, while the glycans are shown as ball-and-sticks. (B) Expanded view of the 3H+109L and 9H+109L epitopes. The V1 region is highlighted by coloring Ala137 in yellow, and the GDIR motif in the V3 region is in red. Heavy chain (HC; C) and light chain (LC; D and E) contacts of gl-NIH45-46 with 426c.TM1ΔV1-3 (PDB accession no. 5IGX) and gl-VRC01 with eOD-GT6 (PDB accession no. 4JPK) superimposed onto the structure of BG505 SOSIP.v4.1-GT1-N137A. Protein backbones are shown as Cα traces, key interacting residues are shown in stick representations (red, oxygen; blue, nitrogen), and yellow dashed lines indicate putative hydrogen bonds (distance < 4 Å). Antibodies: orange, gl-NIH45-46 HC; magenta, gl-NIH45-46 LC; yellow, gl-VRC01 HC; green, gl-VRC01 LC. gp120: blue, BG505 SOSIP.v4.1-GT1; light blue, 426c.TM1ΔV1-3. A and C are based on similar figures in Scharf et al. (2016).

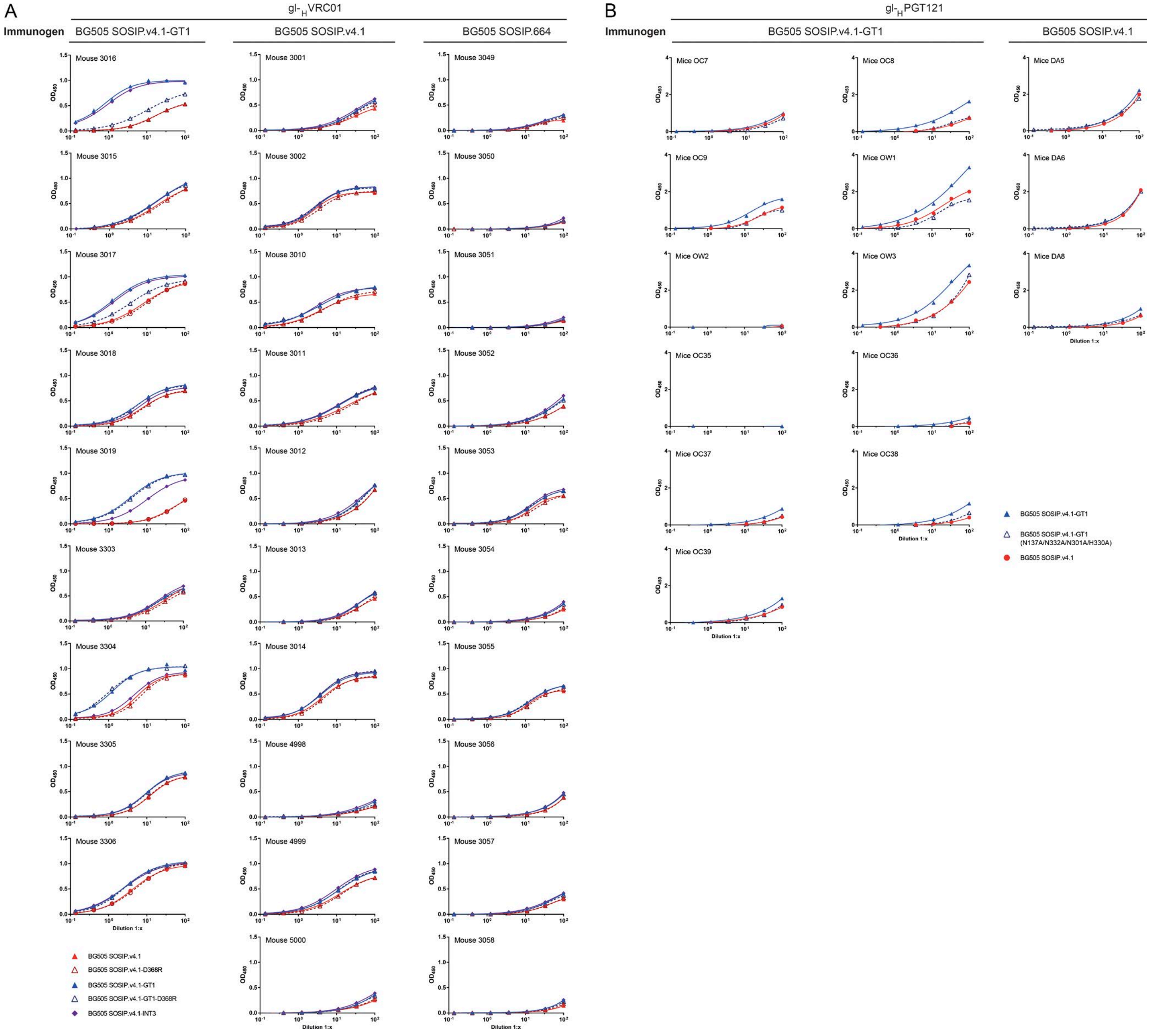

Figure S5. **Env trimer binding of sera derived from immunization of gl$_H$-VRC01 and gl$_H$-PGT121 knock-in mice with different SOSIP trimers (related to Fig. 5, B–E).** (A) Sera (postimmunization 3) from gl$_H$-VRC01 knock-in mice immunized with BG505 SOSIP.v4.1-GT1 (left), SOSIP.v4.1 (middle), or SOSIP.664 (right) trimers were titrated against the indicated trimers by ELISA. The sera were serially diluted in threefold steps, starting from a 1:100 dilution. (B) Sera (postimmunization 3) from gl$_H$-PGT121 knock-in mice immunized with BG505 SOSIP.v4.1-GT1 (left and middle) or SOSIP.v4.1 (right) trimers were titrated against the indicated trimers by ELISA. The sera were serially diluted in threefold steps, starting from a 1:100 dilution.

REFERENCE

Scharf, L., A.P. West, S.A. Sievers, C. Chen, S. Jiang, H. Gao, M.D. Gray, A.T. McGuire, J.F. Scheid, M.C. Nussenzweig, et al. 2016. Structural basis for germline antibody recognition of HIV-1 immunogens. *eLife.* 5:e13783. http://dx.doi.org/10.7554/eLife.13783

Table S1. **Related to Figs. 2, 5 (A and F, left), S3 B, and S7 and Tables S3, S4, and S5. Heavy and light chain sequences of the gl-bNAbs used in this study.**

PG9[1]	VH reverted	QVQLVESGGGVVQPGRSLRLSCAASGFTFSSYGMHWVRQAPGKGLEWVAVISYDGSNKYYADSVKGRFTISRDNSKNTLYLQMNSLRAEDTAVYYCAREAGGPDYRNGYNYYDFWSGYYTYYYMDVWGKGTTVTVSS
	VL reverted	QSALTQPASVSGSPGQSITISCTGTSSDVGGYNYVSWYQQHPGKAPKLMIYEVSNRPSGVSNRFSGSKSGNTASLTISGLQAEDEADYYCSSYTSSSTLVFGGGTKLTVL
PG16[2]	VH reverted	QVQLVESGGGVVQPGRSLRLSCAASGFTFSSYGMHWVRQAPGKGLEWVAVISYDGSNKYYADSVKGRFTISRDNSKNTLYLQMNSLRAEDTAVYYCAREAGGPIWHDDVKYYDFNDGYYNYHYMDVWGKGTTVTVSS
	VL reverted	QSALTQPASVSGSPGQSITISCTGTSSDVGGYNYVSWYQQHPGKAPKLMIYEVSNRPSGVSNRFSGSKSGNTASLTISGLQAEDEADYYCSSYTSSSTLIFGGGTKVTVL
PG145[1]	VH reverted	QVQLVQSGAEVKKPGASVKVSCKASGYTFTSYDINWVRQATGQGLEWMGWMNPNSGNTGYAQKFQGRVTMTRNTSISTAYMELSSLRSEDTAVYYCARGSKHRLRDYFLYNEYGPNYEEWGDYLATLDVWGQGTMVTVSS
	VL reverted	DIVMTQSPLSLPVTPGEPASISCRSSQSLLHSNGYNYLDWYLQKPGQSPQLLIYLGSNRASGVPDRFSGSGSGTDFTLKISRVEAEDVGVYYCMQALQTPWTFGQGTKVEIK
CH01[3]	VH reverted	EVQLVESGGGVVRPGGSLRLSCAASGFTFDDYGMSWVRQAPGKGLEWVSGINWNGGSTGYADSVKGRFTISRDNAKNSLYLQMNSLRAEDTALYYCARGTDYTIDDQGIRYQGSGTFWYFDLWGRGTLVTVSS
	VL reverted	EIVLTQSPGTLSLSPGERATLSCRASQSVSSSYLAWYQQKPGQAPRLLIYGASSRATGIPDRFSGSGSGTDFTLTISRLEPEDFAVYYCQQYGSSPYTFGQGTKVEIK
PGT121[4]	VH reverted*	QVQLQESGPGLVKPSETLSLTCTVSGGSISSYYWSWIRQPPGKGLEWIGYIYYSGSTNYNPSLKSRVTISVDTSKNQFSLKLSSVTAADTAVYYCARTQQGKRIYGVVSFGDYYYYYYMDVWGKGTTVTVSS
	VL reverted	SYVLTQPPSVSVAPGQTARITCGGNNIGSKSVHWYQQKPGQAPVLVVYDDSDRPSGIPERFSGSNSGNTATLTISRVEAGDEADYYCQVWDSSSDHPWVFGGGTKLTVL
1NC9[1]	VH reverted	QVQLVQSGAEVKKPGASVKVSCKASGYTFTSYYMHWVRQAPGQGLEWMGIINPSGGSTSYAQKFQGRVTMTRDTSTSTVYMELSSLRSEDTAVYYCAREDSDFHDGHGHTLRGMFDYWGQGTLVTVSS
	VL reverted	QSVLTQPPSASGTPGQRVTISCSGSSSNIGSNYVYWYQQLPGTAPKLLIYRNNQRPSGVPDRFSGSKSGTSASLAISGLRSEDEADYYCAAWDDSLSGPVFGGGTKLTVL
VRC01[5]	VH reverted*	QVQLVQSGAEVKKPGASVKVSCKASGYTFTGYYMHWVRQAPGQGLEWMGWINPNSGGTNYAQKFQGRVTMTRDTSISTAYMELSRLRSDDTAVYYCARGKNSDYNWDFQHWGQGTLVTVSS
	VL reverted	EIVLTQSPATLSLSPGERATLSCRASQSVSSYLAWYQQKPGQAPRLLIYDASNRATGIPARFSGSGSGTDFTLTISSLEPEDFAVYYCQQYEFFGQGTKLEIK
3BNC60[6]	VH reverted	QVQLVQSGAEVKKPGASVKVSCKASGYTFTGYYMHWVRQAPGQGLEWMGWINPNSGGTNYAQKFQGRVTMTRDTSISTAYMELSRLRSDDTAVYYCARERSDFWDFDLWGRGTLVTVSS
	VL reverted	DIQMTQSPSSLSASVGDRVTITCQASQDISNYLNWYQQKPGKAPKLLIYDASNLETGVPSRFSGSGSGTDFTFTISSLQPEDIATYYCQQYEFIGPGTKVDIK
NIH45-46[7]	VH reverted	QVQLVQSGAEVKKPGASVKVSCKASGYTFTGYYMHWVRQAPGQGLEWMGWINPNSGGTNYAQKFQGRVTMTRDTSISTAYMELSRLRSDDTAVYYCARGKYCTARDYYNWDFQHWGQGTLVTVSS
	VL reverted	EIVLTQSPATLSLSPGERATLSCRASQSVSSYLAWYQQKPGQAPRLLIYDASNRATGIPARFSGSGSGTDFTLTISSLEPEDFAVYYCQQYEFFGQGTKLEIK
12A12[5]	VH reverted	QVQLVQSGAEVKKPGASVKVSCKASGYTFTGYYMHWVRQAPGQGLEWMGWINPNSGGTNYAQKFQGRVTMTRDTSISTAYMELSRLRSDDTAVYYCARDGSGDDTSWHFDPWGQGTLVTVSS
	VL reverted	DIQMTQSPSSLSASVGDRVTITCQASQDISNYLNWYQQKPGKAPKLLIYDASNLETGVPSRFSGSGSGTDFTFTISSLQPEDIATYCAVLEFFGPGTKVEIKRTVAAPSV
PGV19[5]	VH reverted	QVQLVQSGAEVKKPGASVKVSCKASGYTFTGYYMHWVRQAPGQGLEWMGWINPNSGGTNYAQKFQGRVTMTRDTSISTAYMELSRLRSDDTAVYYCARMGAAREWDFQYWGQGTRVLVSS
	VL reverted	ESALTQPASVSGSPGQSITISCTGTSSDVGGYNYVSWYQQHPGKAPKLMIYEVSNRPSGVSNRFSGSKSGNTASLTISGLQAEDEADYYCSSYEFFGGGTKLTVLGQ
CH103[8]	VH reverted	QVQLQESGPGLVKPSETLSLTCTVSGGSISSYYWSWIRQPPGKGLEWIGYIYYSGSTNYNPSLKSRVTISVDTSKNQFSLKLSSVTAADTAVYYCASLPRGQLVNAYFDYWGQGTLVTVSS
	VL reverted	SYELTQPPSVSVSPGQTASITCSGDKLGDKYACWYQQKPGQSPVLVIYQDSKRPSGIPERFSGSNSGNTATLTISGTQAMDEADYYCQAWDSFSTFVFGTGTKVTVL
3BC315[1]	VH reverted	QVQLVQSGAEVKKPGASVKVSCKASGYTFTGYYMHWVRQAPGQGLEWMGWINPNSGGTNYAQKFQGRVTMTRDTSISTAYMELSRLRSDDTAVYYCARPMRPVSHGIDYSGLFVFQFWGQGTMVTVSS
	VL reverted	TGSVTQSALTQPASVSGSPGQSITISCTGTSSDVGSYNLVSWYQQHPGKAPKLMIYEVSKRPSGVSNRFSGSKSGNTASLTISGLQAEDEADYYCCSYANYDKLIFGGGTKLTVLSQPKAAPSVTLFPPS

* The same sequences are used in the knock-in mice as in the binding studies.

1. Sliepen et al., 2015
2. Pancera et al., 2010
3. Bonsignori et al., 2011
4. Escolano et al., 2016
5. Jardine et al., 2013
6. Dosenovic et al., 2015
7. Scharf et al., 2013
8. Liao et al., 2013

Table S2. Related to Fig. 1 (A and B). Neutralization sensitivity of a panel of viruses to the inferred germline versions of PG9, PG16 and PGT145. The TZM-bl cell assay was used to determine the percentage of neutralization at the maximum concentration of antibody. The ACS viruses are clinical isolates, BL035 and Q23 are Env-pseudotyped viruses and BG505 is a molecular clone (see SI Methods). The neutralization assays were performed in triplicate. The mean values, with standard deviations, are shown and ranged by color as indicated. The V2 sequence column shows the relevant sequences of residues 156 to 196 (HxB2 numbering system). The BG505 SOSIP.v4.1-GT1 sequence is indicated by the black arrow, the changes are highlighted in dark grey boxes/white characters, and the 7 amino-acid deletion is indicated by the gray shading over the dashed line. The origins of those changes are indicated by dashed boxes. The R178K change that was taken from elsewhere (Aussedat et al., 2013) is indicated with a shade of gray.

(Table S2 appears separately as an Excel file)

Table S3. Related to Fig. 1 B. Relative binding of a panel of three gl-bNAbs and two bNAbs to BG505 SOSIP.664 variants in a D7324-capture ELISA using supernatants from transfected HEK293T cells.

Modifications introduced to BG505 SOSIP.664			Germline[4]			Mature	
**Stability[2]**	**V1V2-apex gl-bNAb Enhancement**	**CD4bs gl-bNAb enhancement**	**PG9**	**VRC01**	**PGV19**	**2G12**	**PGT145**
-	-	-	-	-	-	+++++	+++++
A316W	-	-	-	-	-	+++++	+++++
A316W E64K	-	-	-	-	-	+++++	+++++
A316W	ΔRSNNSNK[3]	-	+	-	-	+++++	+++++
A316W	ΔRSNNSNK[3]	N462D	+	-	-	+++++	+++++
A316W	ΔRSNNSNK[3]	N276D	+	-	+/-	+++++	+++++
A316W	ΔRSNNSNK[3]	N276D N462D	+	-	+	+++++	+++++
A316W E64K	ΔRSNNSNK[3]	N276D N462D	+	-	+	+++++	+++++
A316W E64K	ΔRSNNSNK[3]	N276D N462D G471S	+	-	++	+++++	+++++
A316W E64K	ΔRSNNSNK[3]	N276D N462D N386D	+	-	++	+++++	+++++
A316W E64K	ΔRSNNSNK[3]	N276D N462D T278R	+	+	+++	+++++	+++++
A316W E64K	ΔRSNNSNK[3]	N276D N462D T278R N386D G471S	+	++	++++	+++++	+++++
A316W E64K	ΔRSNNSNK[3] K169R Y173H S174A R178K V181I Q183P	N276D N462D T278R N386D G471S S199A	++	+++	++++	+++++	+++++
A316W E64K	ΔRSNNSNK[3] K169R Y173H S174A R178K V181I Q183P	N276D N462D T278R N386D G471S S199A	++	+++	++++	+++++	+++++
A316W E64K	ΔRSNNSNK[3] K169R Y173H S174A R178K V181I Q183P G188N N189T 190S	N276D N462D T278R N386D G471S S199A	++++	++++	++++	+++++	+++++

1. Unpurified HEK293T cell culture supernatant.
2. See de Taeye et al. (2015).
3. Seven amino-acid deletion between residues 185e and 190 (HxB2 numbering).
4. See Table S1.

Table S4. Related to Fig. 1 B. Relative binding of panel of three gl-bNAbs and two bNAbs to BG505 SOSIP.664 variants in a Ni-NTA/His-tag capture ELISA using supernatants from transfected HEK293T cells.

Modifications introduced to BG505 SOSIP.664			Germline[4]			Mature	
**Stability[2]**	**V1V2-apex gl-bNAb Enhancement**	**CD4bs gl-bNAb enhancement**	**PG9**	**VRC01**	**PGV19**	**2G12**	**PGT145**
-	-	-	-	-	-	+++++	+++++
A316W	-	-	-	-	-	+++++	+++++
A316W E64K	-	-	-	-	-	+++++	+++++
A316W E64K	ΔRSNNSNK[3]	N276D N462D	+	-	+	+++++	+++++
A316W E64K	ΔRSNNSNK[3]	N276D N462D G471S	+	-	++	+++++	+++++
A316W E64K	ΔRSNNSNK[3]	N276D N462D N386D	+	-	++	+++++	+++++
A316W E64K	ΔRSNNSNK[3]	N276D N462D T278R	+	++	+++	+++++	+++++
A316W E64K	ΔRSNNSNK[3] K169R Y173H S174A R178K V181I Q183P G188N N189T 190S	N276D N462D T278R N386D G471S S199A	+++	++++	++++	+++++	+++++

1. Unpurified HEK293T cell culture supernatant.
2. See de Taeye et al. (2015).
3. Seven amino-acid deletion between residues 185e and 190 (HxB2 numbering).
4. See Table S1.

Table S5. Related to Fig. 1 B. Relative binding of panel of three gl-bNAbs and two bNAbs to BG505 SOSIP.664 variants by Ni-NTA/His-tag capture ELISA using affinity chromatography purified trimers.

Modifications introduced to BG505 SOSIP.664			Mermline[4]			Mature	
Stability[2]	V1V2-apex gl-bNAb Enhancement	CD4bs gl-bNAb enhancement	PG9	VRC01	PGV19	2G12	PGT145
			++	-	-	+++++	++++
A316W	-	-	++	-	-	+++++	+++++
A316W E64K	-	-	+++	-	-	+++++	+++++
A316W E64K	ΔRSNNSNK[1]	N276D N462D	+++	-	ND	+++++	+++++
A316W E64K	ΔRSNNSNK[1]	N276D N462D G471S	+++	+	ND	++++	++++
A316W E64K	ΔRSNNSNK[1]	N276D N462D N386D	+++	+	ND	++++	++++
**A316W E64K**	**ΔRSNNSNK[1]**	**N276D T278R**	+++	+++	+++++	+++++	+++++
A316W E64K	ΔRSNNSNK[1]	N276D T278R N462D	+++	++++	+++++	+++++	+++++
A316W E64K	ΔRSNNSNK[1] K169R Y173H S174A R178K V181I Q183P G188N N189T 190S	N276D N462D T278R N386D G471S S199A	+++++	+++++	+++++	+++++	+++++

ND. Not determined.
1. Trimers purified with affinity chromatography using a PGT145 column.
2. See de Taeye et al. (2015).
3. Seven amino-acid deletion between residues 185e and 190 (HxB2 numbering).
4. See Table S1.

Table S6. Related to Fig. 1 E. Percentage of $Man_{5-9}GlcNAc_2$ glycans (M5-M9) as the proportion of the total glycan population.

Molecule \ %	M5	M6	M7	M8	M9	Sum
**BG505 SOSIPv.4.1-GT1**	7	5	9	22	17	**59**
**BG505 SOSIP.v4.1**	8	5	7	21	23	**64**
**BG505 SOSIP.664**	8	5	8	19	25	**66**

Table S7. Related to Fig. 2 B. SPR analysis of the mature and germline versions of a panel of bNAbs to SOSIP.664 trimer variants.

(Table S7 appears separately as an Excel file)

Table S8. Related to Figs. 3 and 4. X-ray data collection and refinement statistics.

Data collection	BG505 SOSIP.v4.1-GT1-N137A + 9H+109L + 35O22
Beamline	APS 23-ID-D
Wavelength (Å)	1.033
Space group	$P6_3$
Unit cell (Å)	a = b=127.0, c=315.2
Resolution (Å)	50.0-3.2 (3.26-3.20) [a]
Observations	610,919
Unique reflections	47,220 (2,375) [a]
Redundancy	12.9 (13.1) [a]
Completeness (%)	100 (100) [a]
$<I/\sigma_I>$	11.0 (1.0) [a]
R_{sym} [b]	0.17 (1.00) [a]
R_{pim} [c]	0.08 (0.77) [a]
$CC_{1/2}$ [d]	0.89 (0.52)
Refinement statistics	
Resolution (Å)	49.4-3.2
Reflections (work)	44,674
Reflections (test)	2,409
R_{cryst} (%) [e]	23.8[c] (40.4) [a]
R_{free} (%) [f]	26.6[d] (41.8) [a]
Average B-value (Å^2)	
All proteins	126
gp120	106
gp41	115
9H+109L (V_H/V_L)	122
9H+109L (C_L/C_H1)	207
35O22 (V_H/V_L)	122
35O22 (C_L/C_H1)	207
Glycans	99
Wilson B-value (Å^2)	97
RMSD from ideal geometry	
Bond length (Å)	0.010
Bond angles (°)	0.772
Ramachandran statistics (%) [g]	
Favored	94.5
Outliers	0.7
PDB ID	5W6D

[a]Numbers in parentheses refer to the highest resolution shell.

[b]$R_{sym} = \Sigma_{hkl}\Sigma_i \mid I_{hkl,i} - <I_{hkl}> \mid / \Sigma_{hkl}\Sigma_i I_{hkl,I}$, where $I_{hkl,i}$ is the scaled intensity of the ith measurement of reflection h, k, l, $<I_{hkl}>$ is the average intensity for that reflection, and n is the redundancy .

[c]R_{pim} is a redundancy-independent measure of the quality of intensity measurements. $R_{pim} = \Sigma_{hkl} (1/(n-1))^{1/2} \Sigma_i \mid I_{hkl,i} - <I_{hkl}> \mid / \Sigma_{hkl} \Sigma_i I_{hkl,I}$, where $I_{hkl,i}$ is the scaled intensity of the ith measurement of reflection h, k, l, $< I_{hkl} >$ is the average intensity for that reflection, and n is the redundancy.

[d]$CC_{1/2}$ = Pearson Correlation Coefficient between two random half datasets.

[e]$R_{cryst} = \Sigma_{hkl} \mid F_o - F_c \mid / \Sigma_{hkl} \mid F_o \mid \times 100$

[f]R_{free} was calculated as for R_{cryst}, but on a test set comprising 5% of the data excluded from refinement.

[g]Calculated using MolProbity .
